# Mitochondrial genomes of acrodont lizards: timing of gene rearrangements and phylogenetic and biogeographic implications

**DOI:** 10.1186/1471-2148-10-141

**Published:** 2010-05-13

**Authors:** Yasuhisa Okajima, Yoshinori Kumazawa

**Affiliations:** 1 Division of Biological Science, Graduate School of Science, Nagoya University, Furo-cho, Chikusa-ku, Nagoya 464-8602, Japan; 2 Department of Information and Biological Sciences, Graduate School of Natural Sciences, Nagoya City University, 1 Yamanohata, Mizuho-cho, Mizuho-ku, Nagoya 467-8501, Japan

## Abstract

**Background:**

Acrodonta consists of Agamidae and Chamaeleonidae that have the characteristic acrodont dentition. These two families and Iguanidae *sensu lato *are members of infraorder Iguania. Phylogenetic relationships and historical biogeography of iguanian lizards still remain to be elucidated in spite of a number of morphological and molecular studies. This issue was addressed by sequencing complete mitochondrial genomes from 10 species that represent major lineages of acrodont lizards. This study also provided a good opportunity to compare molecular evolutionary modes of mitogenomes among different iguanian lineages.

**Results:**

Acrodontan mitogenomes were found to be less conservative than iguanid counterparts with respect to gene arrangement features and rates of sequence evolution. Phylogenetic relationships were constructed with the mitogenomic sequence data and timing of gene rearrangements was inferred on it. The result suggested highly lineage-specific occurrence of several gene rearrangements, except for the translocation of the tRNA^Pro ^gene from the 5' to 3' side of the control region, which likely occurred independently in both agamine and chamaeleonid lineages. Phylogenetic analyses strongly suggested the monophyly of Agamidae in relation to Chamaeleonidae and the non-monophyly of traditional genus *Chamaeleo *within Chamaeleonidae. *Uromastyx *and *Brookesia *were suggested to be the earliest shoot-off of Agamidae and Chamaeleonidae, respectively. Together with the results of relaxed-clock dating analyses, our molecular phylogeny was used to infer the origin of Acrodonta and historical biogeography of its descendant lineages. Our molecular data favored Gondwanan origin of Acrodonta, vicariant divergence of Agamidae and Chamaeleonidae in the drifting India-Madagascar landmass, and migration of the Agamidae to Eurasia with the Indian subcontinent, although Laurasian origin of Acrodonta was not strictly ruled out.

**Conclusions:**

We detected distinct modes of mitogenomic evolution among iguanian families. Agamidae was highlighted in including a number of lineage-specific mitochondrial gene rearrangements. The mitogenomic data provided a certain level of resolution in reconstructing acrodontan phylogeny, although there still remain ambiguous relationships. Our biogeographic implications shed a light on the previous hypothesis of Gondwanan origin of Acrodonta by adding some new evidence and concreteness.

## Background

Acrodonta is a group of squamate lizards that have the characteristic acrodont dentition. Acrodonta consists of two families, Agamidae and Chamaeleonidae, while Pleurodonta having the pleurodont dentition includes Iguanidae *sensu lato *[1]. These three families are extant related members of the infraorder Iguania [2], containing approximately 1,500 species in the world [1]. They are mainly dendrophilous or ground living, and herbivorous or insectivorous animals. Agamids show a certain level of morphological convergence with iguanids whereas chamaeleonids are morphologically quite unique in various organs, such as eye, tongue, tail, and toe [1]. In contrast to the mainly New World distribution of Iguanidae, extant acrodont families are distributed primarily in tropical or subtropical regions of the Old World [1]. Agamids are widely distributed in Asia (including Indoaustralian Archipelago), Australasia and Africa. Chamaeleonids can be seen in Africa (including islands off East Africa), Madagascar, Middle East, Spain, and South Asia.

Phylogenetic relationships between iguanian lizards have been studied both morphologically [2-9] and molecularly [10-18]. These studies are in agreement with one another with respect to the monophylies of Acrodonta (Agamidae + Chamaeleonidae) and Chamaeleonidae. However, the monophyletic status of both Iguanidae and Agamidae is still under debate and these families have often been treated as metataxa (e.g., [2,3,10]). Within the families Agamidae and Chamaeleonidae, morphological studies identified several groups that nearly correspond to agamid subfamilies [3] and chamaeleonid genera [5]. However, the interrelationships of these relatively deep-branch clades have not been settled yet even by the molecular studies that combined a few genes from mitochondrial and/or nuclear genomes. Collection of more nucleotide sites may help to increase resolution in phylogenetic analyses.

Complete mitochondrial genomes may be a target for this purpose because they provide ample nucleotide sites with straightforward sequencing and analytical procedures (see, e.g., [19]) and because they are relatively free from DNA recombinations and gene duplications/deletions [20], albeit with some criticisms on the close genetic linkages of mitochondrial genes, potential saturation of rapidly evolving sites, and occasional strong positive selections [21]. Vertebrate mitochondrial DNAs (mtDNAs) are 16 - 18 kbp double-stranded circular DNAs, which encode 37 genes for 22 tRNAs, 2 rRNAs, and 13 proteins together with a major noncoding region or control region (CR) that is believed to regulate replication and transcription of the mtDNA [20,22]. The gene organization of these encoded genes and the CR is usually very conservative but, in some groups such as Agamidae, gene rearrangements have been reported [23-25]. Why the evolution of mitochondrial genomes is less conservative in some lineages with respect to rates of changes in the sequence and the gene arrangement is still an open question.

When we started this study, complete or nearly complete mtDNA sequences were known from four agamids [24-27] and one chamaeleonid [28]. Macey et al. [29] later added more than a dozen of chamaeleonid taxa mostly from genus *Chamaeleo*. In this study, we collected additional mitogenomic sequences from taxa that represent major groups of Agamidae and Chamaeleonidae. Using these molecular data, we addressed to clarify 1) frequency and timing of the gene rearrangements, 2) phylogenetic relationships among major acrodont groups, and 3) historical biogeography of acrodont lizards in their higher taxonomic level.

## Results

### Mitogenomic features of Acrodonta

Complete or nearly complete mtDNA sequences of 5 agamid and 5 chamaeleonid species were determined for this study (Table 1). These sequences, together with previously reported mitogenomic sequences for 4 agamids [24-27] and 7 chamaeleonids [28,29], were used to characterize features of acrodontan mitogenomes. The acrodontan mitogenomes contained 37 genes that show clear sequence similarity to corresponding mitochondrial genes from other vertebrates. Anticodon sequences of tRNA genes were identical to those of other vertebrates with an exception.

**Table 1:** Iguanian taxa analyzed for their complete mtDNA sequence in this study

Scientific name	**Accession No**.	mtDNA length (bp)	CR length (bp)	**Voucher No**.	Reference
Family Agamidae					
*Uromastyx benti*	AB114447	16380	990	NSMT-H4670	this study^1^
*Leiolepis guttata*	AB476400	16552	1167	NUM-Az389	this study^1^
*Pogona vitticeps*	AB166795	16751	798	NUM-Az383	Amer and Kumazawa [25]
*Chlamydosaurus kingii*	EF090422	16761	812	-	Ujvari et al. [27]
*Hydrosaurus amboinensis*	AB475096	16129	823	SDNCU-x029	this study
*Calotes versicolor*	AB183287	16670	1504	NUM-Az382	Amer and Kumazawa [24]
*Acanthosaura armata*	AB266452	16544	1463	NSMT-H4595	this study^1^
*Pseudotrapelus sinaitus*	AB262447	16560	1456	-	this study^1^
*Xenagama taylori*	DQ008215	16220	1174	CAS225502	Macey et al. [26]
Family Chamaeleonidae					
*Calumma parsonii*	AB474915	17497	2182	SDNCU-x030	this study
*Trioceros melleri*	AB474916	16832	1521	SDNCU-x031	this study
*Chamaeleo calcaricarens*	EF222195	17451	2189	CAS225435	Macey et al. [29]
*Chamaeleo chamaeleon*	EF222201	17415	2155	CAS217781	Macey et al. [29]
*Chamaeleo calyptratus*	EF222192	17433	2178	MVZ236475	Macey et al. [29]
*Chamaeleo zeylanicus*	EF222191	18923	3665	MVZ248409	Macey et al. [29]
*Chamaeleo monachus*	EF222190	18900	3672	MVZ236484	Macey et al. [29]
*Chamaeleo dilepis*	EF222189	17875	2618	CAS168922	Macey et al. [29]
*Furcifer oustaleti*	AB185326	18021	2785	NUM-Az380	Kumazawa [28]
*Kinyongia fischeri*	AB474917	17400*	-	SDNCU-x032	this study
*Rieppeleon kerstenii*	AB474918	17982*	-	SDNCU-x033	this study
*Brookesia decaryi*	AB474914	17324	2094	SDNCU-x034	this study
Family Iguanidae					
*Anolis cybotes*	AB218960	17853*	-	NSMT-H4587	Okajima and Kumazawa [18]
*Basiliscus vittatus*	AB218883	16948	1562	NSMT-H4588	Okajima and Kumazawa [18]
*Gambelia wislizenii*	AB218884	17563	2181	NSMT-H4589	Okajima and Kumazawa [18]
*Iguana iguana*	AJ278511	16633	1191	-	Janke et al. [32]
*Oplurus grandidieri*	AB218720	17122	1758	NSMT-H4590	Okajima and Kumazawa [18]
*Chalarodon madagascariensis*	AB266748	16851	1493	NSMT-H4591	Okajima and Kumazawa [18]
*Polychrus marmoratus*	AB266749	17743	2371	NSMT-H4592	Okajima and Kumazawa [18]
*Leiocephalus personatus*	AB266739	16681	1316	NSMT-H4593	Okajima and Kumazawa [18]
*Plica plica*	AB218961	17643*	-	NSMT-H4594	Okajima and Kumazawa [18]
*Sceloporus occidentalis*	AB079242	17072	1689	-	Kumazawa [33]

Asterisks for the mtDNA length represent values for nearly complete mitogenomes with imcomplete CR sequences. The length of CR does not include that of tRNA^Pro ^gene for chameleons.

^1^Mitochondrial genome sequences for these taxa were originally determined by Dr. Sayed A. M. Amer and provided for this study through his courtesy.

Abbreviations for museums are: NSMT, National Science Museum, Tokyo; NUM, Nagoya University Museum; SDNCU, Specimen Depository of the Graduate School of Natural Sciences, Nagoya City University; CAS, California Academy of Science; and MVZ, Museum of Vertebrate Zoology, University of California at Berkeley.

The anticodon triplet sequence of mitochondrial tRNA^Pro ^genes is usually 5'-TGG-3' [30]. Thus, an unmodified uridine is likely to be present at the first anticodon base of mature tRNA^Pro^s that are responsible for decoding all four CCN codons [31]. So far, only unmodified uridine in this position is considered to be capable of pairing with all four bases at the third codon positions, while modification of the uridine leads to decoding only partial codon members of the 4-codon boxes [31]. *Calotes versicolor*, a member of Draconinae, had the 5'-TGG-3' anticodon in the tRNA^Pro ^gene. However, *Acanthosaura armata*, another representative of Draconinae, was found to have a 5'-CGG-3' anticodon. This is not a sequencing error, for the same 5'-CGG-3' sequence was found in the second individual of *A. armata *and an individual of *Acanthosaura lepidogaster *(data not shown). At present, we do not know how the CCN codons, all of which appear frequently in protein-coding genes of *A. armata *mtDNA, are decoded by products of this aberrant tRNA^Pro ^gene and/or possibly imported proline tRNAs from cytosol. Unknown modification or editing starting from cytidine at the first anticodon position may be responsible for this.

**Figure 1:**
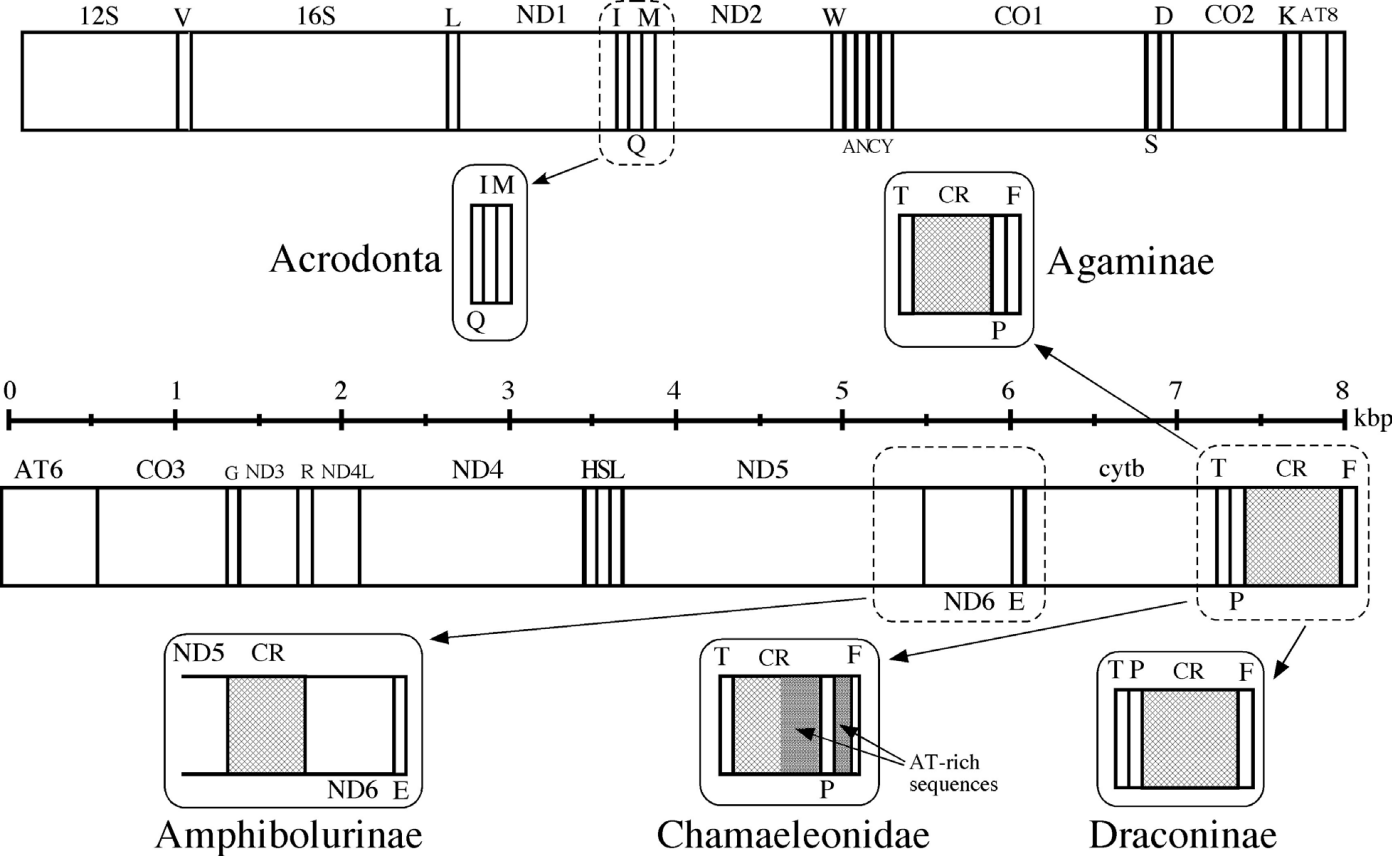
**Mitochondrial genomes of acrodont lizards**. The typical vertebrate mtDNA gene organization that occurs in most major vertebrate groups [22] is linearly shown with columns that approximate sizes of individual genes and the CR, to which changes found in acrodontan mitogenomes are shown. Genes encoded by the heavy strand are shown above the columns, whereas those encoded by the light strand are shown below them. Gene abbreviations used are: 12S, 12S rRNA; 16S, 16S rRNA; ND1-6, NADH dehydrogenase subunits 1-6; CO1-3, cytochrome oxidase subunits I-III; AT6 and AT8, ATPase subunits 6 and 8; cytb, cytochrome *b*; and one-letter codes of amino acids, tRNA genes specifying them.

Figure 1 summarizes gene arrangement changes found in acrodontan mitogenomes. First, all these mitogenomes shared a tRNA gene rearrangement from IQM to QIM [23] that is not present in the iguanid mitogenomes [18,32,33], supporting that this gene rearrangement took place in the ancestral acrodontan lineage after its divergence from Iguanidae [23].

**Figure 2:**
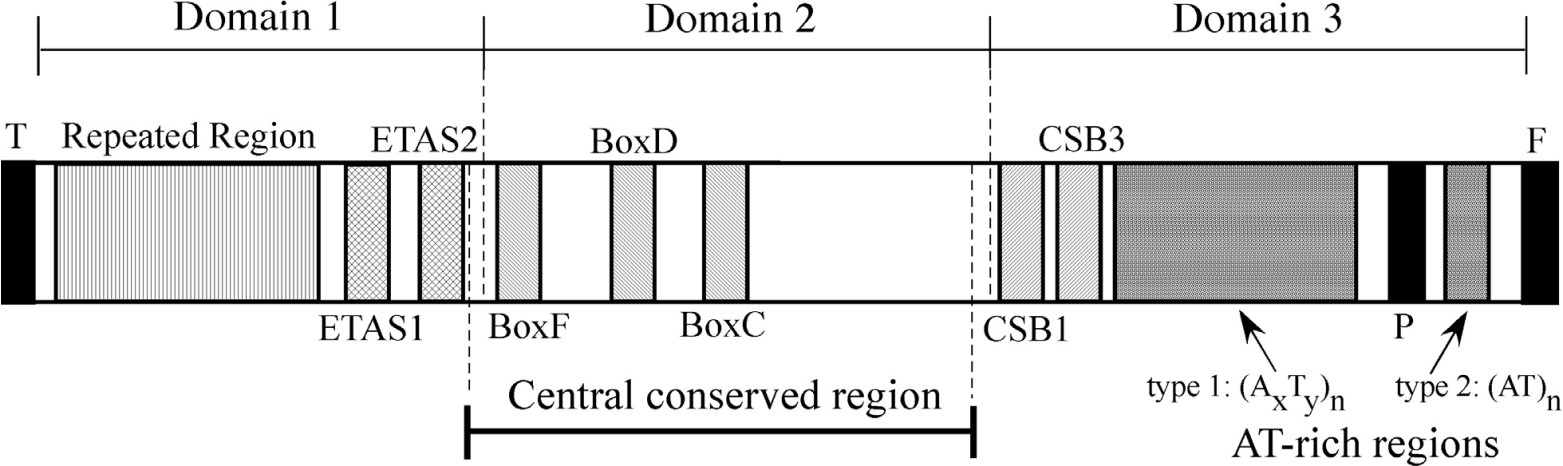
**Features in control regions of chamaeleonid lizards**. The CR is divided into three domains in which occurrence of distinct features is shown: ETAS 1 and 2, extended termination associated sequence 1 and 2; Box C, D and F, central conserved box C, D and F; and CSB 1 and 3, conserved sequence block I and III. Two types of AT-rich sequences that occur in chamaeleonid CRs are: (A_x_T_y_)_n _and (AT)_n_.

Acrodontan mitogenomes show extensive variations in the location and orientation of the tRNA^Pro ^gene, which is typically encoded by the light strand and located between the tRNA^Thr ^gene and the CR in most other vertebrates (Fig. 1). All the chamaeleonids examined possess the tRNA^Pro ^gene at the 3' side of the CR, 200-400 bp 5' to the tRNA^Phe ^gene (see Fig. 2 and Additional File 1). The translocated tRNA^Pro ^gene accompanies two different types of AT-rich regions around it (see below). Among agamids, the tRNA^Pro ^gene was found in the typical location and orientation for representatives from Uromastycinae, Leiolepidinae, Amphibolurinae and Hydrosaurinae. Two representatives from Draconinae (*C. versicolor *and *A. armata*) had an inverted tRNA^Pro ^gene (i.e., the switch of an encoded strand from L to H without changing its relative location to other genes and the CR), which was originally found by Amer and Kumazawa [24]. Finally, the tRNA^Pro ^gene was translocated immediately 5' to the tRNA^Phe ^gene in two representatives from Agaminae (*P. sinaitus *and *X. taylori*)[26]. Thus, these anomalies in the position and orientation of tRNA^Pro ^gene were found in a lineage specific manner. Another example of specific changes in the gene arrangement or features was seen in two representatives from Australasian agamid subfamily Amphibolurinae (*P. vitticeps *and *C. kingii*), in which duplicate CR sequences are inserted between ND5 and ND6 genes [25,27]. On the other hand, the characteristic stem-and-loop structure for the putative light-strand replication origin is known to have disappeared from the WANCY tRNA gene cluster in multiple agamid lineages [34]. Among the 31 iguanian taxa examined, all iguanid and chamaeleonid taxa retained this structure, whereas *Uromastyx benti*, *Hydrosaurus amboinensis *and two amphibolurine agamids did not have it.

Control regions of chamaeleonid mitogenomes were considerably longer than agamid and iguanid counterparts. The average length of the CR was 2506 bp among 10 chameleons, while that was 1132 and 1695 bp, respectively, among 9 agamids and 8 iguanids (Table 1). Figure 2 shows the schematic diagram of the chamaeleonid CRs. As seen for mammals (e.g., [35]), Domain 1 contains ETAS (extended termination associated sequences) elements and tandemly repeated sequences. Domain 2 contains the central conserved region in which conserved boxes C, D and F [36] are identified (Fig. 2 and Additional File 1). Domain 3 contains conserved sequence blocks (CSBs) I and III [37] and two types of AT-rich sequences. The first type, that occurs 5' to the tRNA^Pro ^gene, is characterized by repetition of a stretch of A followed by a stretch of T, (A_x_T_y_)_n_, and the second type found mostly 3' to the tRNA^Pro ^gene (except for *Brookesia decaryi *in which it also occurs 5' to the tRNA^Pro ^gene) is a repetition of an AT sequence, (AT)_n_. Although these two types can also be seen in non-chamaeleonid CRs (see, e.g., [38]), they occur in chamaeleonids in much larger scales. Moreover, chameleon CRs, except for *T. melleri*, were found to have a long array of tandemly repeated sequences 5' to the ETAS regions in Domain 1 (Additional File 1). Taken together, the expanded size of chamaeleonid CRs is primarily due to tandem repetitions in Domain 1 but this is also due to the extensive arrays of the two types of AT-rich sequences in Domain 3.

### Phylogenetic analyses

**Figure 3:**
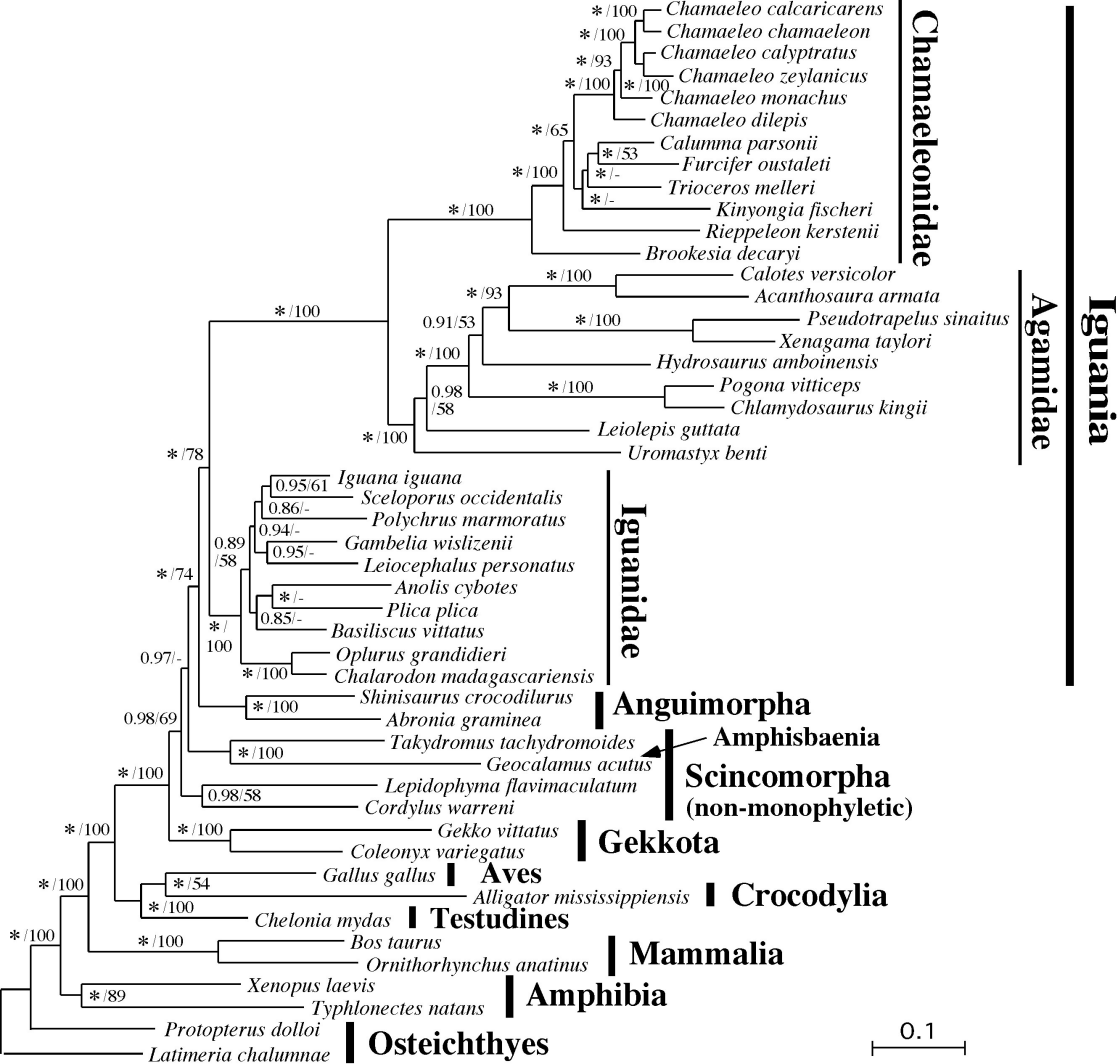
**A Bayesian tree reconstructed using mitogenomic nucleotide sequences**. Values to the left and right of slashes are Bayes-PP and ML bootstrap values (only for those larger than 50%), respectively. An asterisk for the posterior probabilities stands for 1.00. See Table 1 and Additional File 3 for accession numbers of mitogenomic sequences for individual taxa.

Figure 3 shows a Bayesian tree constructed using mitogenomic nucleotide sequences. Non-iguanian parts of the tree were largely consistent with results by previous studies using mitochondrial [28,39] and nuclear [16,40-42] gene sequences. Four saurian infraorders (Iguania, Gekkota, Scincomorpha and Anguimorpha) were recognized but Iguania did not represent the earliest shoot-off among them in sharp contrast with conclusions from morphological studies [2,9]. Iguania consistently clusters with Anguimorpha in these molecular studies including ours. As reported previously [18], Madagascan oplurine iguanids (i.e., *Oplurus grandidieri *and *Chalarodon madagascariensis*) diverged first within Iguanidae *sensu lato*, forming a sister group of other Neotropical iguanids.

Iguanidae forms a sister group of Acrodonta in which Agamidae and Chamaeleonidae are both monophyletic and sister to each other (Fig. 3). These results were supported with 1.00 Bayesian posterior probability (Bayes-PP) and 100% maximum likelihood (ML) bootstrap values. Macey et al. [13] recognized 6 major clades in Agamidae and considered them agamid subfamilies, from which the present study includes 1-2 representative species. Within Agamidae, *Uromastyx benti *(Uromastycinae) diverged first and *Leiolepis guttata *(Leiolepidinae) diverged next (Fig. 3). Uromastycinae and Leiolepidinae have often been considered to be sister to each other, making a basal clade in Agamidae [4,7,43]. Among the other four agamid subfamilies, two representatives from Amphibolurinae diverged first, followed by *Hydrosaurus amboinensis *(Hydrosaurinae) and finally by two representatives from each of Agaminae and Draconinae (Fig. 3).

Twelve representative species that cover all 6 traditional genera of Chamaeleonidae were sampled in this study: *Brookesia*, *Rhampholeon *(now divided into *Rhampholeon *and *Rieppeleon *[44]), *Calumma*, *Furcifer*, *Bradypodion *(now divided into *Bradypodion*, *Kinyongia *and *Nadzikambia *[45]) and *Chamaeleo *(now divided into *Chamaeleo *and *Trioceros *[46]). Our tree (Fig. 3) shows that *Brookesia decaryi *and *Rieppeleon kerstenii *represent basal chamaeleonid lineages but that they are not sister to each other. Our tree also shows non-monophyly of the traditional genus *Chamaeleo*. Six hornless species in the traditional subgenus *Chamaeleo *(now genus *Chamaeleo *[46]) appeared in an entirely different position from horned *Trioceros melleri*. *T. melleri *forms a sister group of *Calumma parsonii *and *Furcifer oustalleti *and all these three taxa cluster with *Kinyongia fischeri *with the exclusion of the 6 hornless *Chamaeleo *species.

Previous studies [16,28,39] suggested highly accelerated molecular evolutionary rates of acrodontan mitochondrial genes. This tendency was conspicuous for almost all lineages of acrodonts and reflected by considerably longer branches for acrodonts as compared to those for iguanids (Fig. 3).

### Divergence times

**Figure 4:**
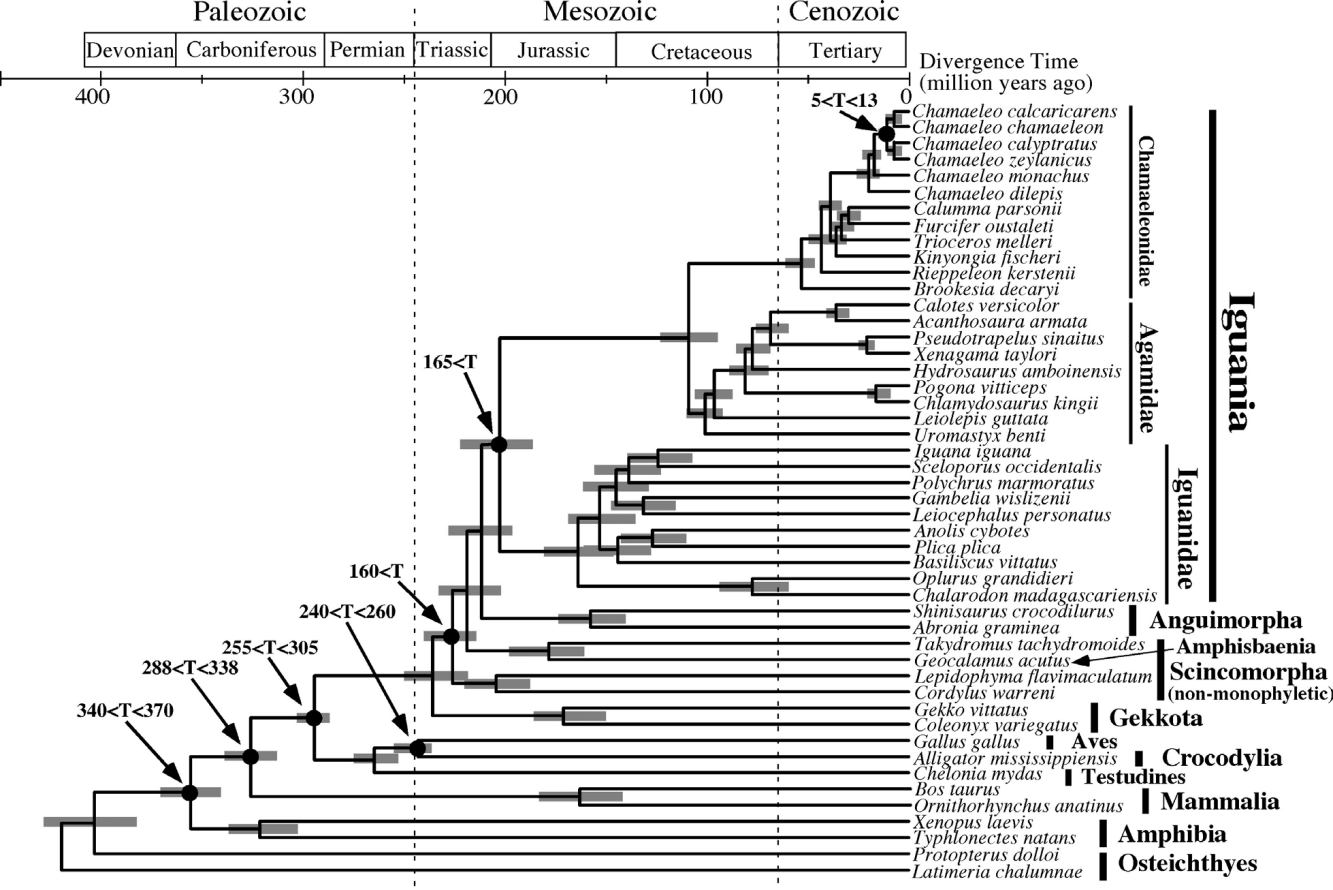
**Divergence times estimated by the relaxed-clock dating method**. Estimated divergence times at individual nodes are shown with their mean and 95% credibility ranges (shaded rectangles). Seven time constraints assumed for the time estimation are indicated at the corresponding nodes (see text for more details).

Figure 4 shows divergence times estimated using the mitogenomic dataset. Although there are not many squamate fossil records that can effectively constrain lower and upper boundaries of divergences [47], we included some available ingroup calibrations inside Squamata in addition to relatively solid fossil-based calibrations outside Squamata. The use of the relaxed-clock method for divergence time estimation [48] seems necessary in light of the heterogeneity of molecular evolutionary rates across lineages as seen by considerably longer and shorter branch lengths from the root to tips (Fig. 3).

The results suggest Triassic-Jurassic [186-220 million years ago (MYA) for the 95% credibility range] divergence of Acrodonta from Iguanidae but much younger mid-Cretaceous (96-122 MYA) divergence into Agamidae and Chamaleonidae (Fig. 4). Agamid subfamilies may have separated during Late Cretaceous times while chamaeleonid radiations into extant genera were estimated to have occurred in the Cenozoic after the K/T boundary. Among the time constraints used in the divergence time estimation, only the 5<T<13 MYA divergence between African and Arabian *Chamaeleo *species was based on a biogeographic assumption. In order to evaluate effects of this ingroup calibration on divergence time estimation, we also conducted the multidistribute analysis without this constraint. The resultant divergence times at various iguanian nodes (data not shown) were very similar to those obtained including this biogeographic constraint (Fig. 4), supporting its congruence with other older time constraints based on fossil evidence.

Recent molecular dating with multiple nuclear genes (reviewed in [42]), though based on different dating methods and time constraints, suggested somewhat younger dates for the divergence of Acrodonta from Pleurodonta (~145 MYA) and the separation into Agamidae and Chamaeleonidae (~85 MYA). However, there is some topological difference between the studies (e.g., non-monophyly of Agamidae in [41]) and several different analyses could provide at most 20 million years of confidence interval in each side of the best time estimate [41]. Thus, the nuclear time estimates for the Agamidae-Chamaeleonidae split may partly overlap our mitochondrial estimate (96-122 MYA, Fig. 4).

## Discussion

### Structural evolution of acrodontan mitogenomes

In the present study, mitogenomic sequences were collected from major representative lineages of Acrodonta to provide an opportunity to compare mitogenomic structures among three iguanian families. Iguanid mitogenomes were very conservative with no gene rearrangements. They also appear to have evolved much more slowly than agamid and chamaeleonid counterparts as judged from relatively short branch lengths within Iguanidae (Fig. 3). On the other hand, acrodontan (especially agamid) mitogenomes have an entirely different tendency with occasional gene rearrangements and increased molecular evolutionary rates.

**Figure 5:**
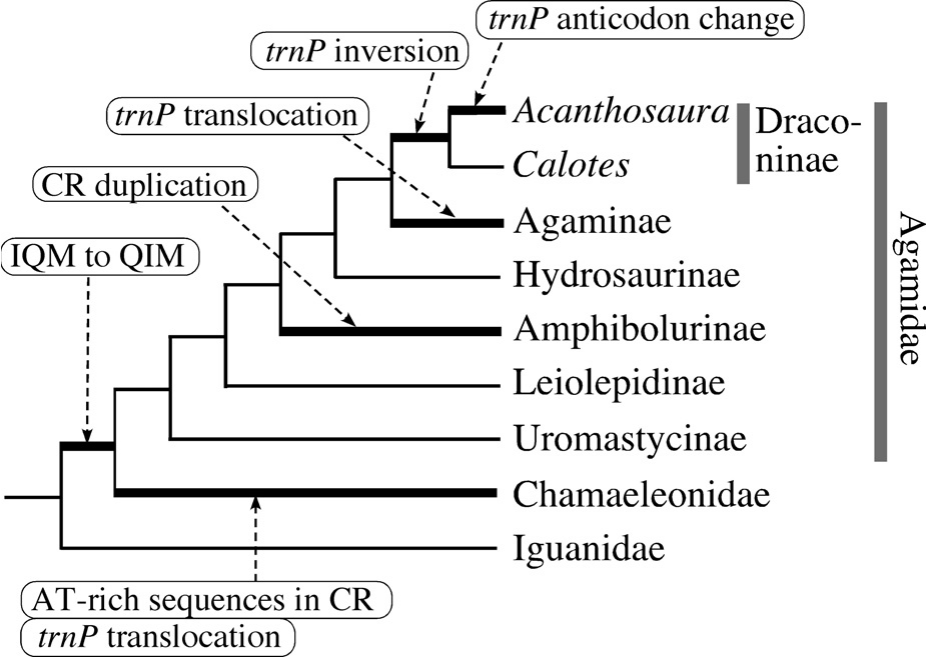
**Occurrence of mitogenomic structural changes in acrodont lizards**. Lineages on which individual changes occurred were supposed by the parsimony criterion based on the phylogenetic framework (Fig. 3) and distributions of gene arrangements in extant species. See Fig. 1 for actual changes in the gene arrangements. The anticodon change in the tRNA^Pro ^gene is TGG to CGG (see text).

In Fig. 5, possible lineages for the mitogenomic reorganizations described here are mapped along the phylogeny based on the parsimony criterion. Although agamid mitogenomes have several examples of gene rearrangements, most of them can be assigned to specific lineages without multiple parallel changes, supporting the rarity and less homoplasious nature of mitochondrial gene rearrangements [22]. The only possible homoplasious change is the translocation of the tRNA^Pro ^gene from the 5' to 3' side of the CR in both Agaminae and Chamaeleonidae. However, the tRNA^Pro ^genes in these taxa are placed in a somewhat different genomic context; i.e., neighboring sequences composed of two types of AT-rich sequences are always present in the chamaeleonids (Fig. 2). This supports that translocations of the tRNA^Pro ^gene in agamines and chamaeleonids resulted from independent events. Moreover, if this translocation had occurred once in a common ancestral lineage of Agaminae and Chamaeleonidae, multiple reversals to the original location of the tRNA^Pro ^gene must be assumed in several agamid lineages, which seems unlikely (Fig. 5). Gene rearrangements around the CR have been shown to occur independently in multiple lineages by the canonical duplication-and-deletion mechanism [22].

To the best of our knowledge, very few studies [38,49] have characterized structural organization and evolution of CRs in lizard mitogenomes. The present study shows that a region encompassing Boxes C, D and F retains a notable similarity among acrodonts and even among diverse groups of vertebrates (Additional File 1). On the other hand, acrodontan CRs do not seem to conserve CSB II in Domain 3 and agamids do not even retain CSB III (Additional File 1). This is in sharp contrast with CRs of several iguanids, gekkonids and lacertids which conserve all three members of CSBs (Additional File 1; [38]), presenting another evidence for more conservative nature of iguanid mitogenomes than acrodontan counterparts.

### Phylogenetic relationships

As outlined earlier, acrodontan phylogeny was originally reconstructed with morphological data, which has been evaluated with molecular data using some mitochondrial and nuclear gene sequences. The present study addressed this issue using the hitherto longest molecular dataset (9,386 bp). As a trade-off of gaining the large number of sites, we had to somewhat sacrifice the depth of taxon sampling. It is thus important to assess monophylies of individual groups in order to interpret our results with respect to subfamilial or generic interrelationships.

Fortunately, previous molecular studies using a number of taxa but less sites provided strong evidence for a number of clades in Agamidae and Chamaeleonidae. For example, Honda et al. [12], Macey et al. [13] and Amer and Kumazawa [50] strongly, in terms of high bootstrap or other tree-support values, suggested monophylies of Uromastycinae, Amphibolurinae, Draconinae and Agaminae within Agamidae. The remaining subfamilies Leiolepidinae and Hydrosaurinae contain limited numbers of extant species and the monophyly of some leiolepidine species was also strongly supported [13]. In addition, the occurrence of subfamily-specific gene rearrangements (Fig. 5; [24,25]) is consistent with the monophylies of the corresponding groups. Within Chamaeleonidae, recent molecular studies using multiple gene sequences [46,51] supported the monophylies of two of the traditional six genera (i.e., *Brookesia and Furcifer*). Remaining traditional genera *Chamaeleo*, *Calumma, Bradypodion *and *Rhampholeon *may each be an assemblage of a few monophyletic groups [14,15,46,51] but no definitive conclusion has been obtained on their phylogenetic relationships.

Our mitogenomic tree (Fig. 3) strongly supports the monophyly of Agamidae relative to Chamaeleonidae (1.00 Bayes-PP and 100% ML-BP values). This is not an artifact due to, e.g., the long branch attraction because all acrodontan lineages appear to possess accelerated molecular evolutionary rates relative to iguanids (Fig. 3). Morphological analyses (e.g., [7,10]) and some molecular ones (e.g., [16,41,52]) did not support the agamid monophyly while other molecular studies (e.g., [13,17,53]) did. Although Agamidae has been regarded as a metataxon under the tentative assumption of its monophyly [2,3,10], this no longer seems necessary in light of our strong molecular evidence on the agamid monophyly.

Mitogenomic data provided agamid subfamilial interrelationships with strong tree-support values in general (Fig. 3). These relationships are consistent with the most parsimonious tree obtained by Macey et al. [13] using ~1,500 bp mitochondrial gene sequences. However, the traditional morphological view tended to unite *Uromastyx *and *Leiolepis *into a basal clade (e.g., [3]). The Kishino-Hasegawa test (data not shown) suggested that this sister relationship of *Uromastyx *and *Leiolepis *is unlikely, though not rejectable (*p *= 0.275). The sister group relationship of Agaminae and Draconinae is common between morphological [3] and molecular (Fig. 3) results.

Our mitogenomic tree (Fig. 3) was consistent with other molecular studies [14,46] with respect to the most basal divergence of genus *Brookesia *and the subsequent divergence of the *Rhampholeon *+ *Rieppeleon *group. However, this phylogenetic relationship was not clear in Townsend and Larson [15] who used ~1,500 bp mitochondrial gene sequences for phylogenetic inference. We conducted Bayesian analyses using combined mitochondrial gene sequences that were used by Raxworthy et al. [14] and Townsend and Larson [15] and the results (data not shown) also supported the most basal divergence of *Brookesia *as shown in Townsend et al. [51]. Although *Brookesia *(Madagascan leaf chameleons) and *Rhampholeon *(African leaf chameleons) were once grouped into a common subfamily Brookesiinae [5], another morphological study based on osteological characters [6] suggested the basal divergence of *Brookesia *alone. Taken together, but primarily based on our mitogenomic phylogeny (Fig. 3), we conclude that the Madagascan *Brookesia *represents the earliest shoot-off of extant chameleons.

The monophyly of traditional genus *Chamaeleo *was strongly suggested by morphological analyses based on distinct synapomorphies (e.g., four rotulae in a hemipenis) and it was subsequently supported by a molecular study [14]. However, another molecular study [15] found separate occurrence of its two subgenera (*Chamaeleo *and *Trioceros*) in the chamaeleonid phylogeny, albeit with little statistical evaluation for their non-monophyly. The most recent molecular study [46] showed stronger evidence for the separation of *Chamaeleo *and *Trioceros*, proposing their elevation to distinct genera. Our mitogenomic analyses (Fig. 3) showed that *Trioceros melleri *is placed distinctly from 6 representatives from *Chamaeleo*. The Kishino-Hasegawa test rejected (*p *= 0.017) the best tree obtained by constraining the *Chamaeleo *+ *Trioceros *monophyly (Tree 5 in Table 2, see also Additional file 2). The more conservative Shimodaira-Hasegawa test (Table 2) did not reject this tree but its probability was very low (*p *= 0.137), supporting the formal elevation of *Chamaeleo *and *Trioceros *to distinct genera [46].

**Table 2:** Comparison of different phylogenetic hypotheses within Chamaeleonidae

Tree	log L	difference	S.E.	p-KH^1^	p-SH^2^
Tree1	-182540.58	0	best	1.000 +	1.000 +
Tree2	-182640.62	100.04	23.7	0.000 -	0.001 -
Tree3	-182594.41	53.83	20.57	0.006 -	0.030 -
Tree4	-182637.55	96.97	29.64	0.000 -	0.002 -
Tree5	-182610.7	70.12	23.27	0.001 -	0.001 -
Tree6	-182575.68	35.1	16.09	0.017 -	0.137 +

^1^Probabilities by the Kishino-Hasegawa test

^2^Probabilities by the Shimodaira-Hasegawa test

Values with a minus mean 'rejective' in the 5% level

Tree1 : Bayesian tree from this study (Fig. 3)

Tree2 : Topology consistent with Klaver and Böhme [5]

Tree3 : Topology consistent with Raxworthy et al. [14]

Tree4 : Topology consistent with Townsend and Larson [15]

Tree5 : Bayesian tree reconstructed using a combined data set from Raxworthy et al. [14] and Townsend and Larson [15]

Tree6 : Bayesian tree reconscructed with the mitogenomic data set by constraining the monophyly of *Chamaeleo *+ *Trioceros*

See Additional File 2 for Newick representations for each tree.

Together with results on testing some specific hypotheses derived from previous morphological and molecular analyses (Table 2), mitogenomic data seem to provide a certain level of resolution on the chamaeleonid phylogeny. To the best of our knowledge, clustering of *Furcifer *with a group of *Calumma *containing *C. parsonii *(Fig. 3) was not suggested by previous studies. Nor was clustering of these two taxa with *Trioceros *(Fig. 3). Evaluation of these new relationships, which did not receive strong bootstrap supports (Fig. 3), awaits further taxon sampling of mitogenomic and/or nuclear gene data.

### Historical biogeography

**Figure 6:**
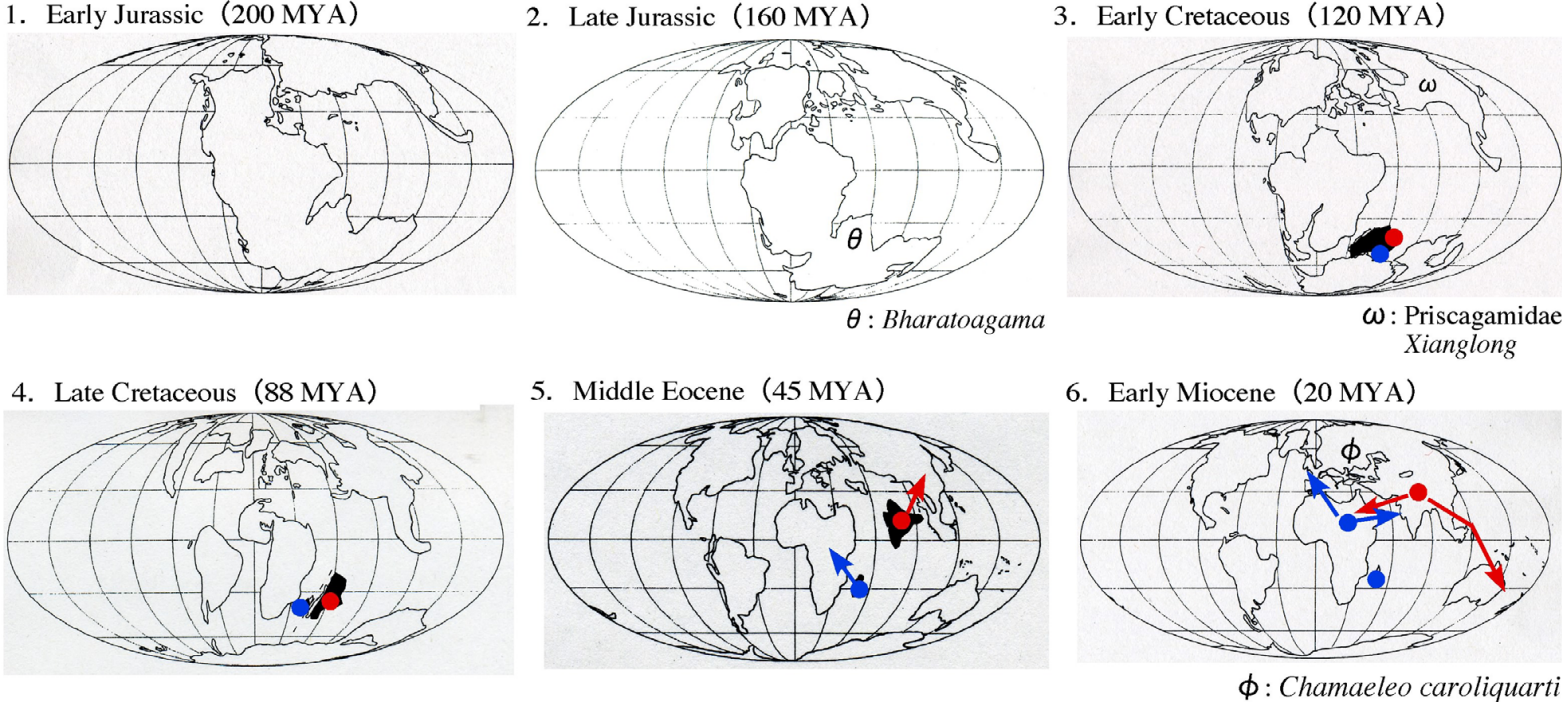
**The historical biogeography of acrodont lizards based on the molecular, paleontological and geological evidence**. Paleogeographical maps at six different times [63] are shown on which a hypothesis on the origin and migration pathways for agamids (red) and chamaeleonids (blue) is illustrated. The earliest fossil records for acrodonts and chameleons are, respectively, Early-Middle Jurassic (165 - 200 MYA) *Bharatoagama *from the Kota Formation of India [61] and Miocene (~26 MYA) *Chamaeleo caroliquarti *from Bohemia [64]. Acrodont fossils of Priscagamidae are found from Aptian-Albian (100 - 120 MYA) and Campanian (~80 MYA) Central Asia and Mongolia [43,54,55]. Another acrodont fossil of a gliding lizard *Xianglong *is found from Early Cretaceous of China [56].

Previous studies on the historical biogeography of Acrodonta did not necessarily postulate the monophylies of Agamidae and Iguanidae. Thus, terms such as the origins of Iguania, Acrodonta and Agamidae have been confusingly used. This study provided strong evidence for the monophylies of Agamidae and Iguanidae, with which previous biogeographic hypotheses can be reevaluated. Here, we discuss the acrodontan biogeography based on molecular, paleontological and geological evidence without *a priori *assumption of vicariance or dispersal (see Fig. 6).

There have been two major hypotheses on the origin of Acrodonta, i.e., where the most recent common ancestor of Agamidae and Chamaeleonidae was, either Laurasian or Gondwanan. Occurrence of acrodontan priscagamid (and even pleurodont iguanian) fossils from mid-late Cretaceous of Asia (Fig. 6.3) led some researchers to hypothesize Laurasian (more specifically Central Asian or Mongolian) origin of Iguania and Acrodonta (e.g., [54,55]). A recent report of a gliding acrodont lizard (*Xianglong*) from Early Cretaceous of China [56] may also support this idea. Extant agamid lizards are distributed primarily in Eurasia but some occur in Australasia and Africa. Recent molecular phylogeny ([12,25,50,57]; but see [13,58]) is consistent with a view that extant Agamidae originated from Asia and that some descendant lineages (e.g., Amphibolurinae and Uromastycinae) dispersed to Australasia and Africa during Cenozoic times when they were geographically connected to or in close proximity to Eurasia. On the other hand, there is good agreement in Gondwanan (more specifically Madagascan or African) origin of extant chamaeleonids [5,6,14,59]. Taken together, the Laurasian origin of Acrodonta requires the long-distance transmarine dispersal of chamaeleonid ancestors from Eurasia to Madagascar/Africa.

Gondwanan origin of Iguania was proposed by Estes [60] based on some fossil evidence and the basal divergence of Iguania from Scleroglossa, which is not tenable by recent molecular phylogeny. Macey et al. [13] used molecular phylogeny to advocate the Gondwanan origin of Acrodonta and proposed that major acrodontan lineages diverged vicariantly and/or migrated to the Northern Hemisphere by plate tectonics (i.e., collision of Indian subcontinent or other Gondwanan land blocks to Eurasia). Although a recent molecular study [17] further supported the out-of-India radiation of a subfamily Agaminae, other molecular studies [25,50,57] questioned the Gondwanan vicariance or multiple northward migrations for at least some lineages (e.g., Amphibolurinae and Uromastycinae).

More recently, some fossil evidence shed a light on this issue. *Bharatagama *from Early-Middle Jurassic Kota Formation of India (Fig. 6.2) represents the oldest record of early acrodont iguanians [61]. This fossil record may support the Gondwanan origin of acrodonts [47,61]. If acrodonts did originate from Gondwanaland, it is consistent with the likely Gondwanan origin of Iguanidae *sensu lato *([18]; refs. therein) but ancestors of extant agamids, which were postulated to be in Asia (see above), may need to have migrated from the Southern to Northern Hemisphere by transmarine dispersal.

The present study (Fig. 4) suggested that Agamidae and Chamaeleonidae are each monophyletic and that they diverged from each other in the mid-Cretaceous (96-122 MYA) although independent molecular dating using nuclear genes suggested somewhat younger dates around 85 MYA [42]. Okajima and Kumazawa [18] previously showed that the appreciable gap in the estimated divergence time of oplurine iguanids between mitochondrial [18] and nuclear [62] gene sequences could be due to multiple factors, such as differences in the tree topology and time constraints assumed for each study, the intrinsic data property of mitochondrial and nuclear sequences, and relatively poor squamate fossil records [47] that can be used to constrain ingroup squamate divergences precisely.

In spite of this somewhat low precision of time estimation, the molecular dating results (Fig. 4; [42]) consistently suggest that Agamidae and Chamaeleonidae were separated after the Middle-Late Jurassic break-up of Pangea into Laurasia and Gondwanaland (Fig. 6.2) and when the latter two supercontinents were further fragmented by plate tectonics [63]. Geological data suggest that India and Madagascar were drifted from Gondwanaland in the Early Cretaceous (120-130 MYA)(Fig. 6.3) and separated from each other in the Late Cretaceous (~90 MYA)(Fig. 6.4)[63]. Then, India moved northward and accreted to Eurasia from the latest Cretaceous to Eocene (Fig. 6.5)[63].

Assuming Gondwanan origin of Acrodonta, the molecular dating results (96-122 MYA from Fig. 4 and ~85 MYA from [42] for the divergence of Agamidae and Chamaeleonidae) are consistent with a view that Agamidae vicariantly diverged from Chamaeleonidae on the India/Madagascar landmass. It may be further hypothesized that Agamidae migrated to Eurasia on the drifting Indian subcontinent (Fig. 6.5) while Chamaeleonidae was left within Madagascar and its descendants migrated to Africa over Mozambique Channel and later to Eurasia (Fig. 6.6). Molecular dating (Fig. 4) suggested that extant chamaeleonid genera diverged during Cenozoic times. Some of them are distributed exclusively in Madagascar (*Furcifer*, *Calumma *and *Brookesia*) while the others are distributed in Africa (*Rhampholeon*/*Rieppeleon *and *Bradypodion*/*Kinyongia*/*Nadzikambia*) or in Africa + Eurasia (*Chamaeleo*/*Trioceros*). Because Africa and Madagascar had been clearly separated in the Cenozoic [63], generic radiations of chamaeleonids cannot be associated with Gondwanan vicariance. As previous authors postulated [14,15], chamaeleonids are likely to have experienced transmarine dispersal over Mozambique Channel multiple times. To the best of our knowledge, the oldest certain fossil record of Chamaeleonidae is *Chamaeleo caroliquarti *from western Bohemia [64]. Africa had long been isolated from other continents but connected to Eurasia in the Miocene [63]. Thus, occurrence of this fossil in the Miocene of Europe is consistent with the above-mentioned biogeographic explanation.

Molecular dating (Fig. 4) also suggested that extant agamid subfamilies diverged from each other in the Late Cretaceous. If Agamidae did migrate to Eurasia on India as hypothesized above, the dating result suggests that subfamilial radiations of agamids occurred on the drifting Indian subcontinent. This may sound somewhat unlikely but this is not impossible under the assumption that a number of ancient agamid lineages radiated, dispersed locally in Asia, and became extinct. *Vastanagama susani *and *Tinosaurus indicus *from the Early Eocene of India are known as the earliest certain agamid fossils in South Asia [65].

Then, how can this hypothesis based on Gondwanan origin of extant acrodont groups reconcile with the early Cretaceous *Xianglong *and mid-late Cretaceous priscagamid acrodonts from Laurasian sites (Central Asia and Mongolia)? *Xianglong *and priscagamids are stem acrodont lizards which are unlikely to be nested within extant agamids but their exact phylogenetic positions are not yet known [56,66]. Therefore, they probably diverged from a long branch of stem acrodonts 110-200 MYA (see Fig. 4). These extinct groups may be Laurasian relics of acrodont lizards which had diverged from Gondwanan acrodonts (i.e., direct common ancestors of extant agamids and chamaeleonids) before Pangean break-up into Laurasia and Gondwanaland. Alternatively, they may simply have derived from the Gondwanan ancestors by transmarine dispersal.

## Conclusions

In the present study, mitogenomic sequences collected from major acrodontan lineages were analyzed in comparison with iguanid counterparts. We detected distinct modes of mitogenomic evolution among iguanian families. Agamidae was highlighted in including a number of lineage-specific mitochondrial gene rearrangements and Chamaeleonidae was found to have a much longer CR sequences. Although mitogenomes of these two families appear to evolve at accelerated rates, they still retain certain conserved features in CR sequences.

Our mitogenomic data provided a certain level of resolution in reconstructing acrodontan phylogeny, although there still remain ambiguous relationships. The strong support for the agamid monophyly should influence taxonomic treatment of the metataxon Agamidae and the suggested separation of *Trioceros *from *Chamaeleo *would support elevation of these taxa to distinct genera [46]. More resolution in unsettled acrodontan phylogenies should be sought by sequencing and comparing considerably longer regions of nuclear genomes than ever.

In this study acrodontan biogeography was reevaluated mostly in favor of Gondwanan origin of Acrodonta, which was originally proposed by Estes [60] and Macey et al. [13]. We suggest that the whole extant Agamidae may have migrated to Eurasia with India. However, we do not agree to the possibility that all or most of agamid subfamilies were introduced to Eurasia, Australasia or Africa by the accretion of Gondwanan plates at different times [13].

Finally, we should carefully state that molecular data and our arguments presented herein do not strictly rule out the Laurasian origin of Acrodonta [54,55] under the assumption that the long-distance transmarine dispersal of chamaeleonid ancestor(s) from Laurasian sites to Madagascar/Africa was possible by unknown geological settings. Further efforts in testing these hypotheses, including ours, by diverse approaches should be encouraged in future.

## Methods

### Samples

Animal samples except for *Pseudotrapelus sinaitus *were obtained from local animal dealers as dead specimens, identified for their species name, and registered to public museums or the Specimen Depository of a university (Table 1). Samples for *P. sinaitus *were collected at Bir Abraq, Egypt. Total DNA was extracted from a tiny amount of tissue samples either with a DNeasy Tissue kit (Qiagen) or by the procedure of Asahida et al. [67]. When multiple individuals were available for the same species, we chose an individual having the best DNA quality for sequencing the entire mitogenome. We then used another individual for sequencing short mtDNA regions to confirm that mtDNA sequences from the second individual are identical or nearly identical to those from the first individual. This is a test for the reliability and reproducibility of our experiments.

### Mitochondrial DNA sequencing

Determination of complete mtDNA sequences was carried out essentially as described previously [19]. Several pairs of reptile-oriented primers [19] were used to amplify and sequence different gene segments of mtDNAs. Species-specific primers (data not shown) were then designed in these regions for amplifying longer parts of the mtDNA by the long PCR using LA-Taq or Z-Taq (Takara, Inc). We typically covered the entire mtDNA by a few overlapping long PCR products. The long-PCR products were used as templates for nested PCR amplifications using the various reptile-oriented primers with Z-Taq, giving rise to overlapping 0.6-1.3 kbp products. The complete mtDNA sequences were unambiguously determined by sequencing and assembling these shorter PCR products. For some of the species, the CR contained extensive tandem repeats and we could not sequence a part of the CR (see Table 1).

### Gene identification and data alignment

Protein gene sequences were translated into amino acid sequences with the vertebrate mitochondrial genetic code [36]. The starting and ending sites of these genes were determined by comparison with the corresponding genes from other vertebrates and in consideration of the possible creation of the stop codon by polyadenylation. Transfer RNA genes were identified based on their secondary structure models [30]. Ranges of the small and large rRNA genes were tentatively identified by tRNA genes that surround them. The major noncoding region was regarded as the CR as it contains CSBs [37]. In this way, all 37 genes and the CR were identified for each of the new mtDNA sequences.

Nucleotide sequences for each of the 37 genes were aligned among the newly sequenced 10 taxa and 39 other vertebrates (see Table 1 and Additional File 3 for their taxon names and accession numbers). The MacClade alignment files for each of the 37 genes can be obtained from authors upon request. We used a script written by Mr. Pierre Jonniaux and Dr. Takamasa Suzuki, with which individual genes of the reported mitogenomic sequences were automatically taken from the databases into a fasta file. This facilitated quick and accurate sequence manipulations. Note that *Kinyongia fischeri *mtDNA has recently been sequenced by Macey et al. [29] independently and that we used our *K. fischeri *mtDNA sequence for subsequent analyses. Also note that *Brookesia superciliaris *mtDNA sequence reported by Macey et al. [29] was not used due to its incompleteness (~12 kbp). The outgroup taxa were selected from one or two representatives from major vertebrate groups, such as Amphibia, Mammalia, Aves, Gekkota and Anguimorpha, except for possibly non-monophyletic Scincomorpha (3 representatives). We omitted some taxa (e.g., snakes and dibamids) that have not been placed in the squamate phylogeny securely when estimated with complete mitogenomic sequences [21,28,39,68].

Protein gene sequences were aligned first for their amino acid sequences with the aid of ClustalX [69], with which nucleotide sequences were automatically aligned using the 'Import NBRF Protein Alignment' function of MacClade 4 (Sinauer Associates, Inc). Transfer RNA and rRNA genes were aligned with the aid of their secondary structure models [30,70]. Nucleotide sequences of the first and second codon positions of 12 heavy-strand-encoded protein genes (except for the light-strand-encoded ND6 gene due to its deviated nucleotide and amino acid compositions), 22 tRNA genes, and 2 rRNA genes were concatenated for 31 iguanians and 18 non-iguanian vertebrates. At the level of deep-branch phylogenies of this study, nucleotides at third codon positions clearly accumulate considerable multiple hits (data not shown) and they were removed from phylogenetic analyses. Because the mitochondrial genome of the tuatara lacks three genes including the largest protein-coding gene for ND5 [71], phylogenetic analyses were primarily conducted without the tuatara after removing all gap-containing sites (48 taxa, 9,386 sites in total).

### Phylogenetic analyses

The Bayesian method was mainly used for constructing phylogenetic relationships. For the Bayesian analyses, MrBayes v3.12 [72] was used with the GTR+I+G model selected by MrModeltest 2.2 [73] after separating the data into four partitions (first codon positions, second codon positions, rRNA positions and tRNA positions). Four chains were run simultaneously by Markov chain Monte Carlo (MCMC) process until the ASDSF (average standard deviation of split frequency) index [72] becomes less than 0.01 (2,500,000 generations). After the initial one fourth of the generations were discarded based on the stationarity of the MCMC process at this stage, 18,750 trees were collected from every 100 generations. The 50% majority -rule consensus tree was created from this tree pool and the Bayes-PP values (the frequency of a specific phylogenetic relationship in the sampled tree population) were obtained as a statistical measure for resultant phylogenetic relationships.

We conducted several phylogenetic analyses using different representative taxa from each major group of vertebrates to confirm that taxon selection in the outgroup does not basically change ingroup acrodont relationships (data not shown). As one of such analyses, the Bayesian tree reconstructed with another taxon, the tuatara (Additional File 4), showed the same acrodont relationships as in Fig. 3 although a polytomy occurred with respect to subfamilial relationships of Iguanidae *sensu lato*. We also tested some different partition schemes including the one categorizing protein genes into four different enzyme complexes [68] to confirm that our phylogenetic tree was not affected by such partition scheme (data not shown).

Bootstrap probabilities were assessed by the ML method using GARLI0.96b8 [74] with the GTR+I+G model. The same dataset as used for the Bayesian analysis was supplied without partitioning and 500 replications were carried out. Statistical evaluation of alternative phylogenetic hypotheses was done with TREE-PUZZLE 5.2 [75] using the Kishino-Hasegawa [76] and Shimodaira-Hasegawa [77] tests. The GTR+I+G model and its parameters optimized by MrModeltest 2.2 were used.

### Estimation of divergence times

Divergence times were estimated with the multidistribute program [48] by assuming a topological relationship thus obtained but without assuming the strict molecular clock. The program PAML [78] was first used to optimize parameters for the F84 nucleotide substitution model and the gamma distribution for 8 categories for each of the four partitions separately. The estbranches/multidivtime programs were then run by the Bayesian MCMC method to provide posterior distributions of divergence times at each internal node. Several parameters set to run the MCMC analyses were: numsamps, 10,000; sampfreq, 100; burnin, 100,000; rttm and rttmsd, 4.3; rtrate and rtratesd, 0.05; and brownmean and brownmeansd, 0.2. The coelacanth (*Latimeria chalumnae*) was used as an outgroup in this analysis. We conducted the divergence time estimation with different settings for taxon samplings (e.g., more sparse sampling from Iguanidae and non-iguanians), time constraints (i.e., addition/deletion of some of the seven time constraints used) and some parameters (e.g., brownmean/brownsd and bigtime) to confirm that results were essentially the same.

Similar time constraints for divergence times as used by Okajima and Kumazawa [18] were employed. They are based on relatively reliable fossil-based time constraints for several representative nodes: Amphibia vs. Amniota, 340<T<370 MYA; Synapsida vs. Sauropsida (Reptiles + Aves), 288<T<338 MYA; Archosauria vs. Lepidosauria, 255<T<305 MYA; Aves vs. Crocodylia, 240<T<260 MYA; Acrodonta vs. Pleurodonta, 165<T MYA; and origin of Scincomorpha, 160<T MYA. Refer to Okajima and Kumazawa [18] and Kumazawa [28] for references that justify these time constraints. In addition to these time constraints, we also included a time constraint for a shallower divergence between African and Arabian chameleons within genus *Chamaeleo*. The time constraint for this divergence (5<T<13 MYA) was based on the biogeographic assumption that they diverged vicariantly by the expansion of Red Sea [29] (see [50] for geological evidence for this time range).

## Authors' contributions

YO contributed to sequencing mitochondrial genomes. YO and YK analyzed the data and wrote the manuscript. Both authors read the manuscript and agreed to its publication.

## Supplementary Material

Additional file 1 Alignment of control region sequences from iguanians and other vertebrates. Click here for file

Additional file 2 **Topological relationships assumed for the Kishino-Hasegawa and Shimodaira-Hasegawa tests in table**2. Click here for file

Additional file 3 Non-iguanian outgroup taxa analyzed for their complete mtDNA sequence. Click here for file

Additional file 4 A Bayesian tree reconstructed using mitogenomic nucleotide sequences by including the tuatara and retaining gap-containing sites (49 taxa, 9689 sites). Click here for file
