# Supplementary material for: Mitochondrial genomes of acrodont lizards: timing of gene rearrangements and phylogenetic and biogeographic implications
Source: BMC Evol Biol. 2010 May 13;10:141. doi: 10.1186/1471-2148-10-141 (PMC2889956; doi:10.1186/1471-2148-10-141)
Supplement: Additional file 1 — Alignment of control region sequences from iguanians and other vertebrates. [file 1471-2148-10-141-S1.PDF]

Alignment of control region sequences from iguanians and other vertebrates.

Tandemly repeated regions in Domain 1 are not shown. CR sequences for the ETAS regions in Domain 1, as well as those in Domains 2 and 3 are aligned. Accession numbers for the sequences are the same as in Table 1 and Additional File 2, except for the following: *Fringilla montifringilla* (Brambling, Aves), U76251; *Carduelis chloris* (European greenfinch, Aves), U56076; *Bos taurus* (cow, Mammalia), J01394; *Felis catus* (cat, Mammalia), U20753; *Glis glis* (fat dormouse, Mammalia), Y11137; and *Homo sapiens* (human, Mammalia), J01415.

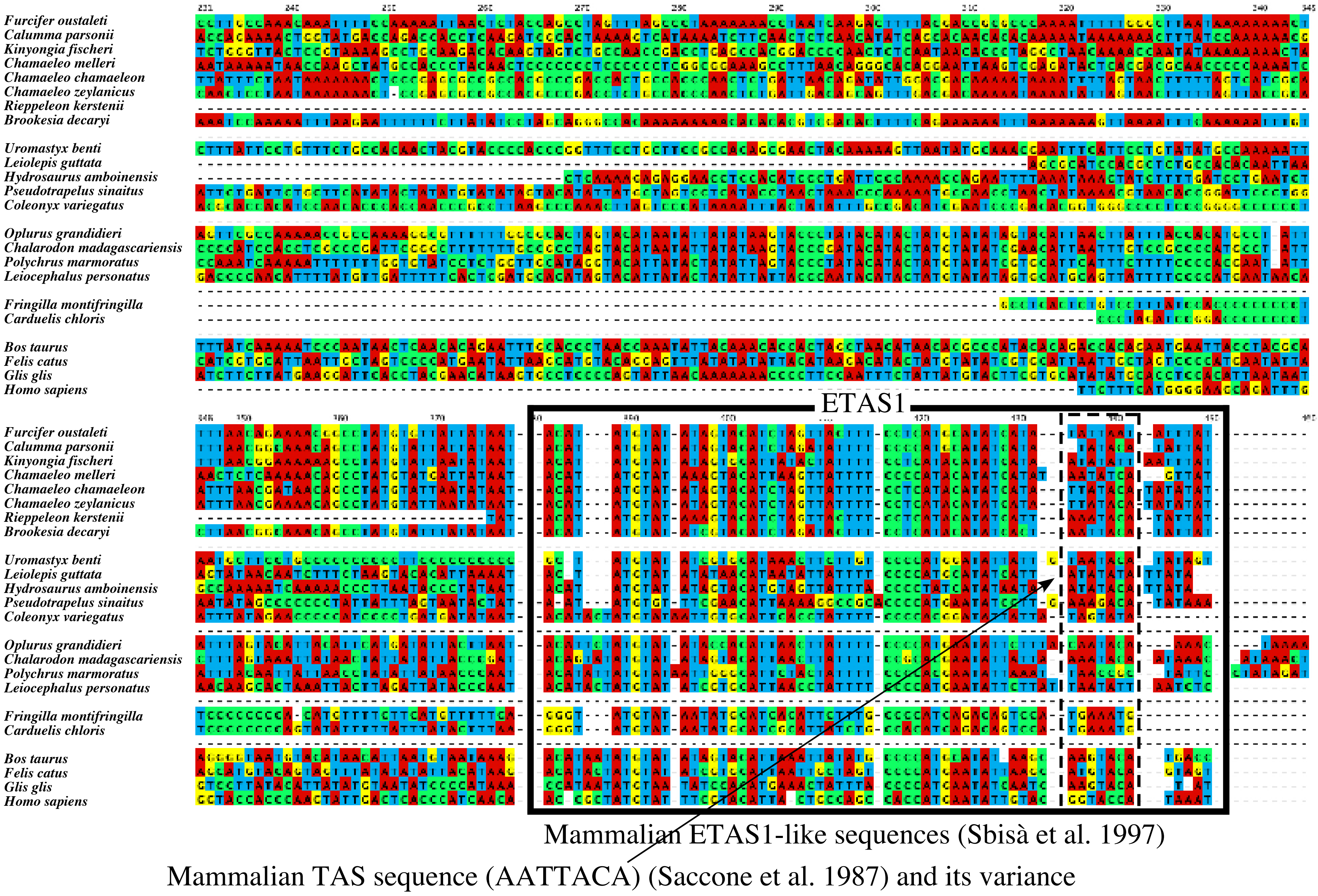
Furcifer oustaleti
Calumma parsonii
Kinyongia fischeri
Chamaeleo melleri
Chamaeleo chamaeleon
Chamaeleo zeylanicus
Rieppeleon kerstenii
Brookesia decaryi
Uromastyx benti
Leiolepis guttata
Hydrosaurus amboinensis
Pseudotrapelus sinaitus
Coleonyx variegatus
Oplurus grandidieri
Chalarodon madagascariensis
Polychrus marmoratus
Leiocephalus personatus
Fringilla montifringilla
Carduelis chloris
Bos taurus
Felis catus
Glis glis
Homo sapiens
ETAS1
Mammalian ETAS1-like sequences (Sbisà et al. 1997)
Mammalian TAS sequence (AATTACA) (Saccone et al. 1987) and its variance

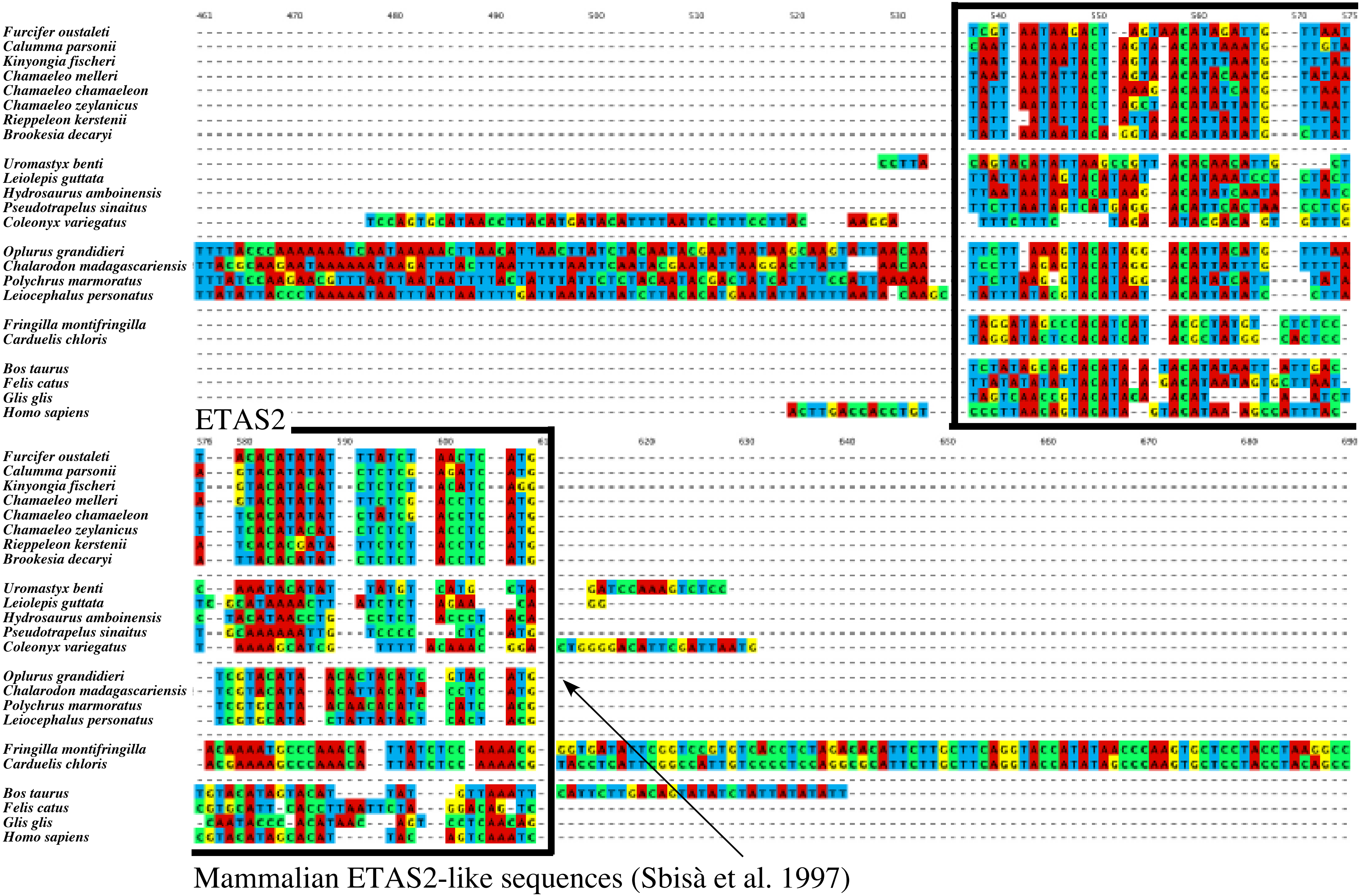
Furcifer oustaleti
Calumma parsonii
Kinyongia fischeri
Chamaeleo melleri
Chamaeleo chamaeleon
Chamaeleo zeylanicus
Rieppeleon kerstenii
Brookesia decaryi
Uromastyx benti
Leiolepis guttata
Hydrosaurus amboinensis
Pseudotrapelus sinaitus
Coleonyx variegatus
Oplurus grandidieri
Chalarodon madagascariensis
Polychrus marmoratus
Leiocephalus personatus
Fringilla montifringilla
Carduelis chloris
Bos taurus
Felis catus
Glis glis
Homo sapiens
ETAS2
Mammalian ETAS2-like sequences (Sbisà et al. 1997)

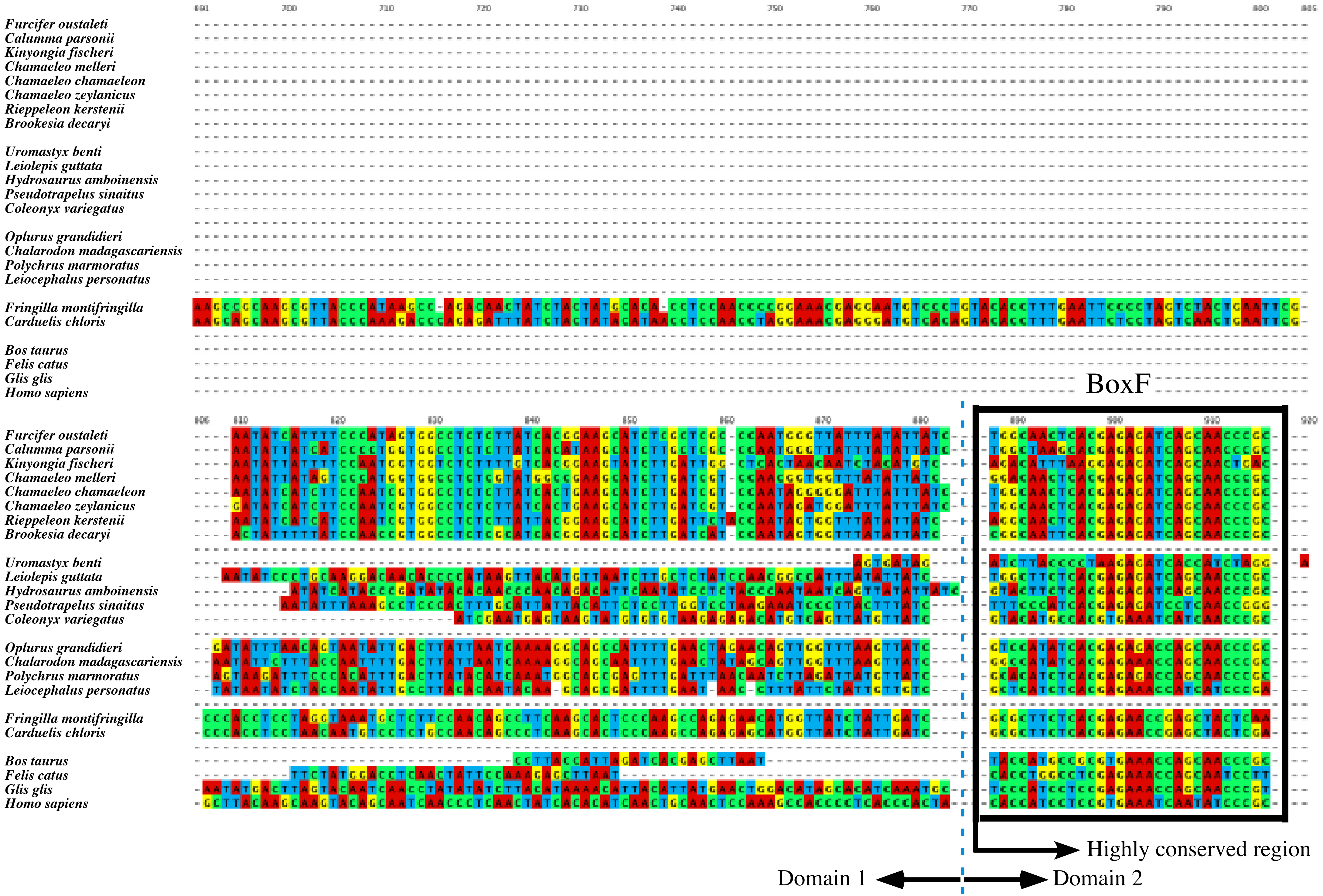
Furcifer oustaleti
Calumma parsonii
Kinyongia fischeri
Chamaeleo melleri
Chamaeleo chamaeleon
Chamaeleo zeylanicus
Rieppeleon kerstenii
Brookesia decaryi
Uromastyx benti
Leiolepis guttata
Hydrosaurus amboinensis
Pseudotrapelus sinaitus
Coleonyx variegatus
Oplurus grandidieri
Chalarodon madagascariensis
Polychrus marmoratus
Leiocephalus personatus
Fringilla montifringilla
Carduelis chloris
Bos taurus
Felis catus
Glis glis
Homo sapiens
BoxF
Highly conserved region
Domain 1
Domain 2

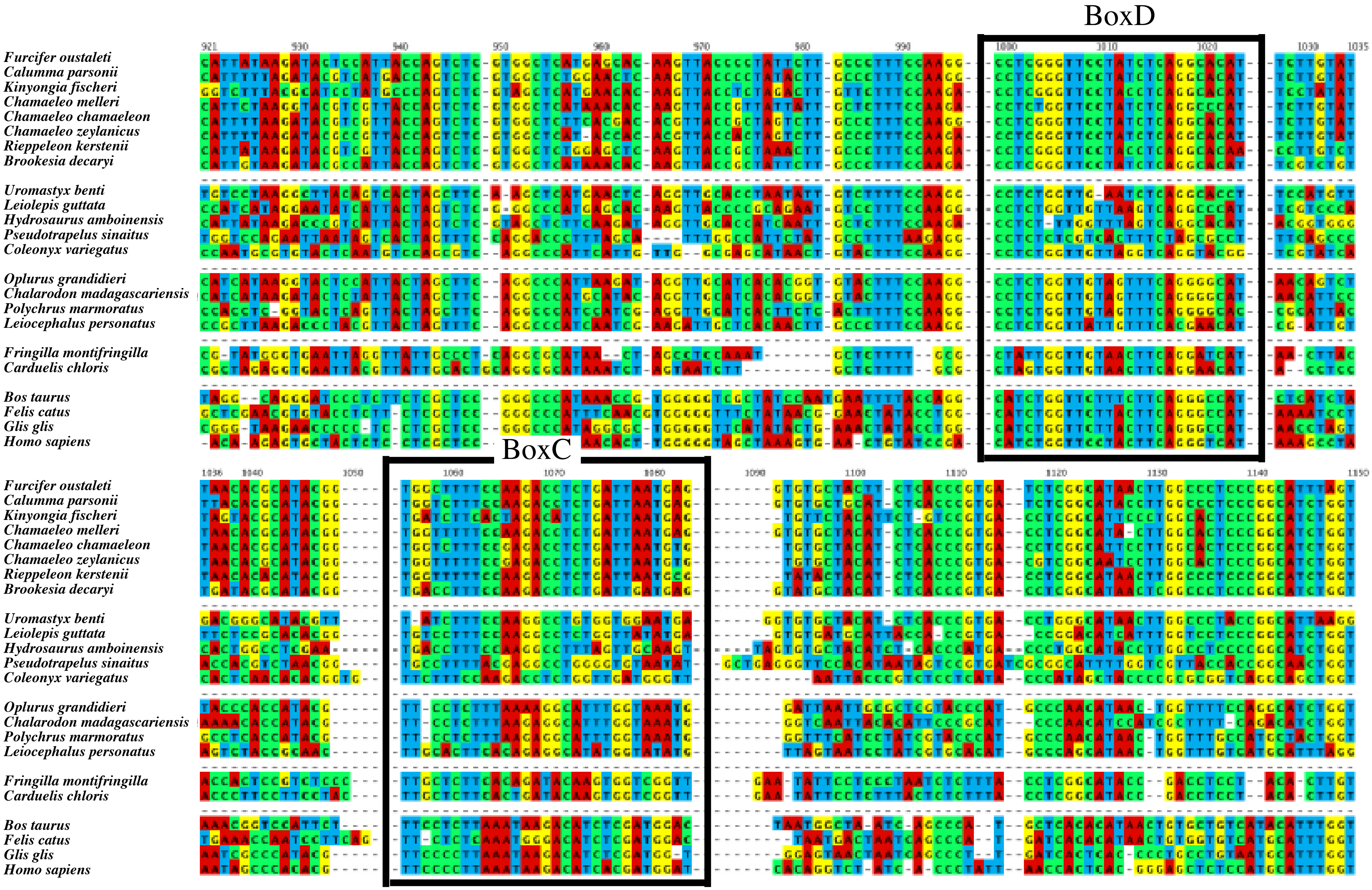
BoxD
BoxC
Furcifer oustaleti
Calumma parsonii
Kinyongia fischeri
Chamaeleo melleri
Chamaeleo chamaeleon
Chamaeleo zeylanicus
Rieppeleon kerstenii
Brookesia decaryi
Uromastyx benti
Leiolepis guttata
Hydrosaurus amboinensis
Pseudotrapelus sinaitus
Coleonyx variegatus
Oplurus grandidieri
Chalarodon madagascariensis
Polychrus marmoratus
Leiocephalus personatus
Fringilla montifringilla
Carduelis chloris
Bos taurus
Felis catus
Glis glis
Homo sapiens

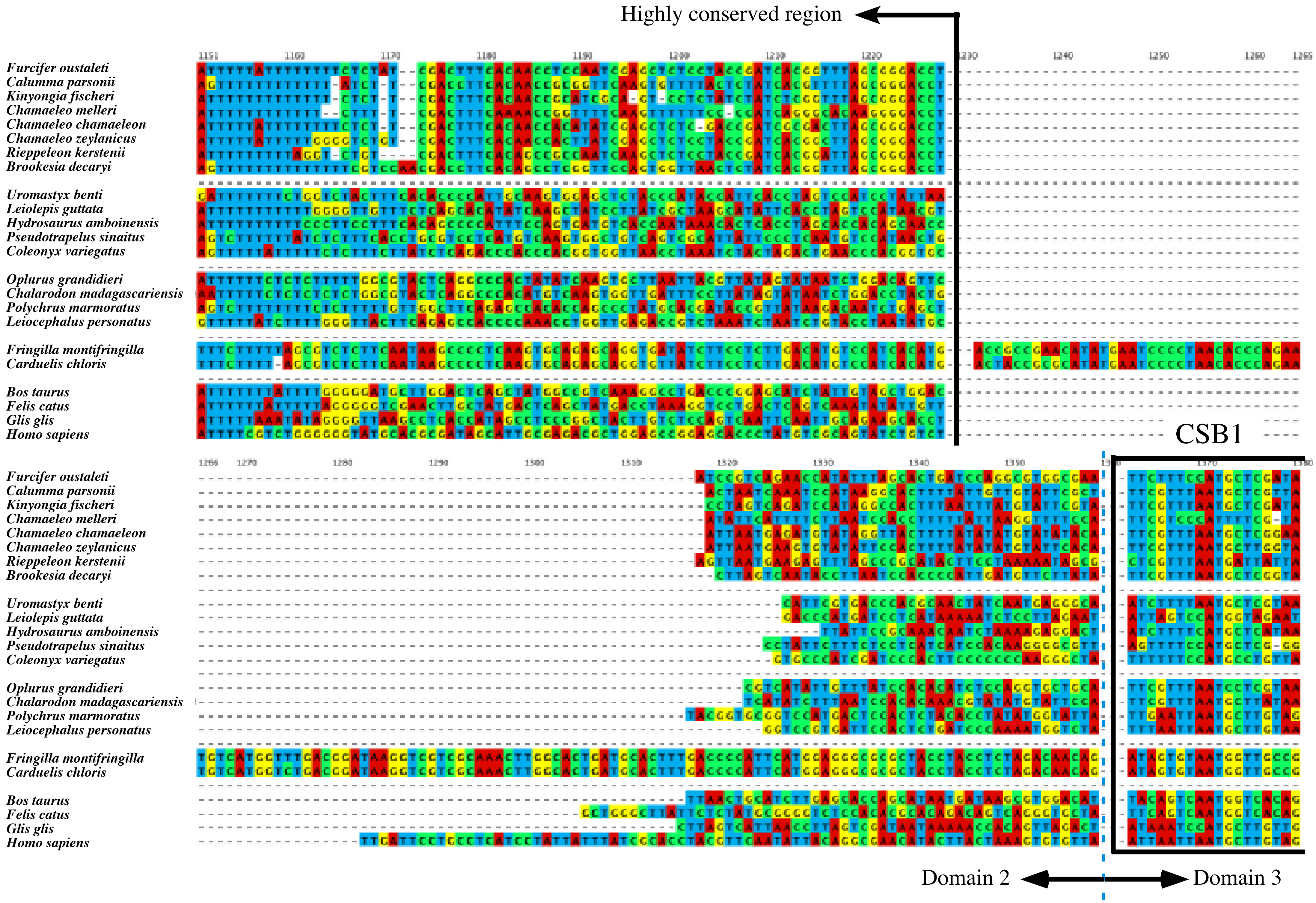
Highly conserved region
Furcifer oustaleti
Calumma parsonii
Kinyongia fischeri
Chamaeleo melleri
Chamaeleo chamaeleon
Chamaeleo zeylanicus
Rieppeleon kerstenii
Brookesia decaryi
Uromastyx benti
Leiolepis guttata
Hydrosaurus amboinensis
Pseudotrapelus sinaitus
Coleonyx variegatus
Oplurus grandidieri
Chalarodon madagascariensis
Polychrus marmoratus
Leiocephalus personatus
Fringilla montifringilla
Carduelis chloris
Bos taurus
Felis catus
Glis glis
Homo sapiens
CSB1
Domain 2
Domain 3

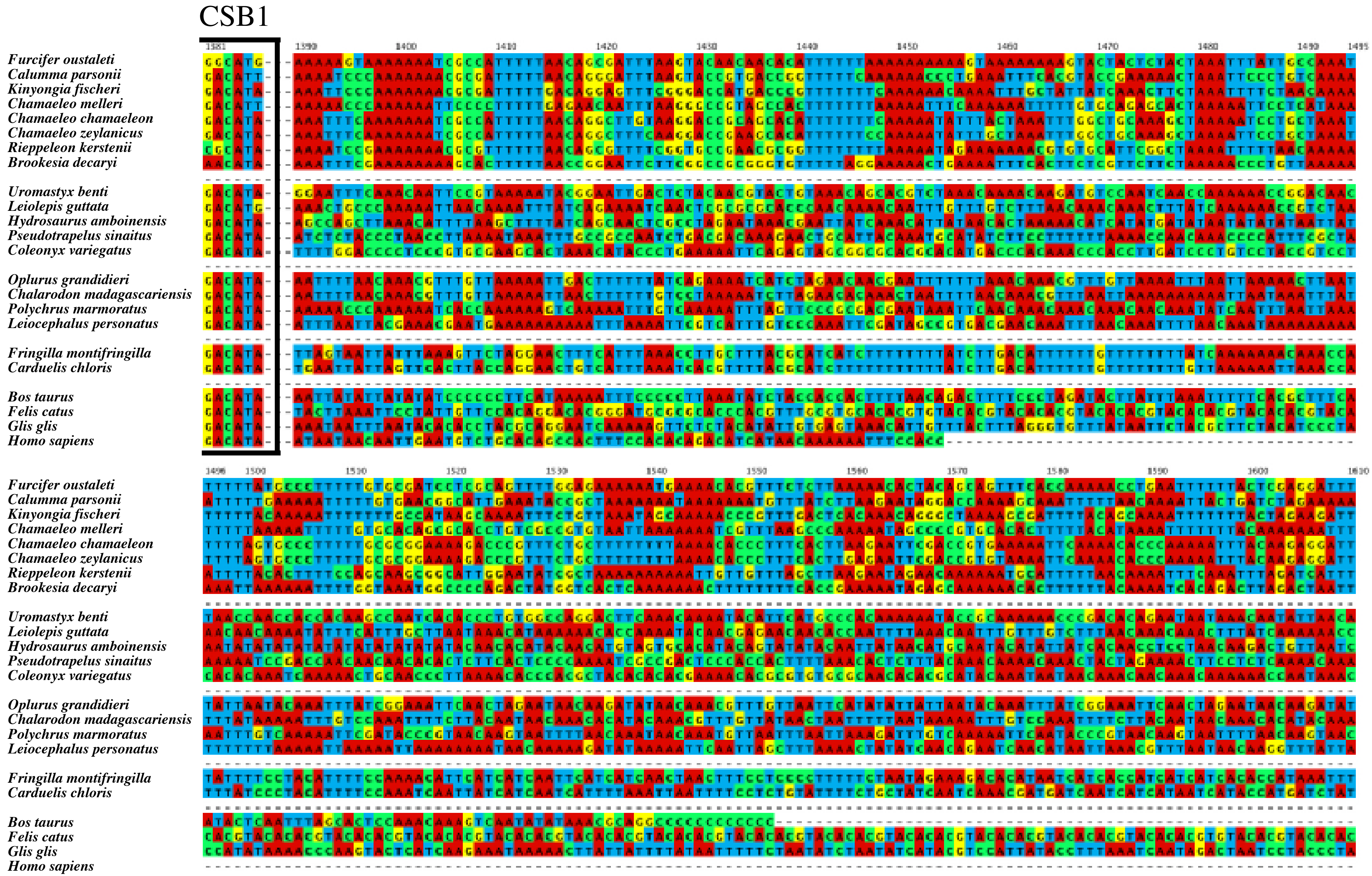
CSB1
1381
1390
1400
1410
1420
1430
1440
1450
1460
1470
1480
1490
1495
1496
1500
1510
1520
1530
1540
1550
1560
1570
1580
1590
1600
1610
Furcifer oustaleti
Calumma parsonii
Kinyongia fischeri
Chamaeleo melleri
Chamaeleo chamaeleon
Chamaeleo zeylanicus
Rieppeleon kerstenii
Brookesia decaryi
Uromastyx benti
Leiolepis guttata
Hydrosaurus amboinensis
Pseudotrapelus sinaitus
Coleonyx variegatus
Oplurus grandidieri
Chalarodon madagascariensis
Polychrus marmoratus
Leiocephalus personatus
Fringilla montifringilla
Carduelis chloris
Bos taurus
Felis catus
Glis glis
Homo sapiens

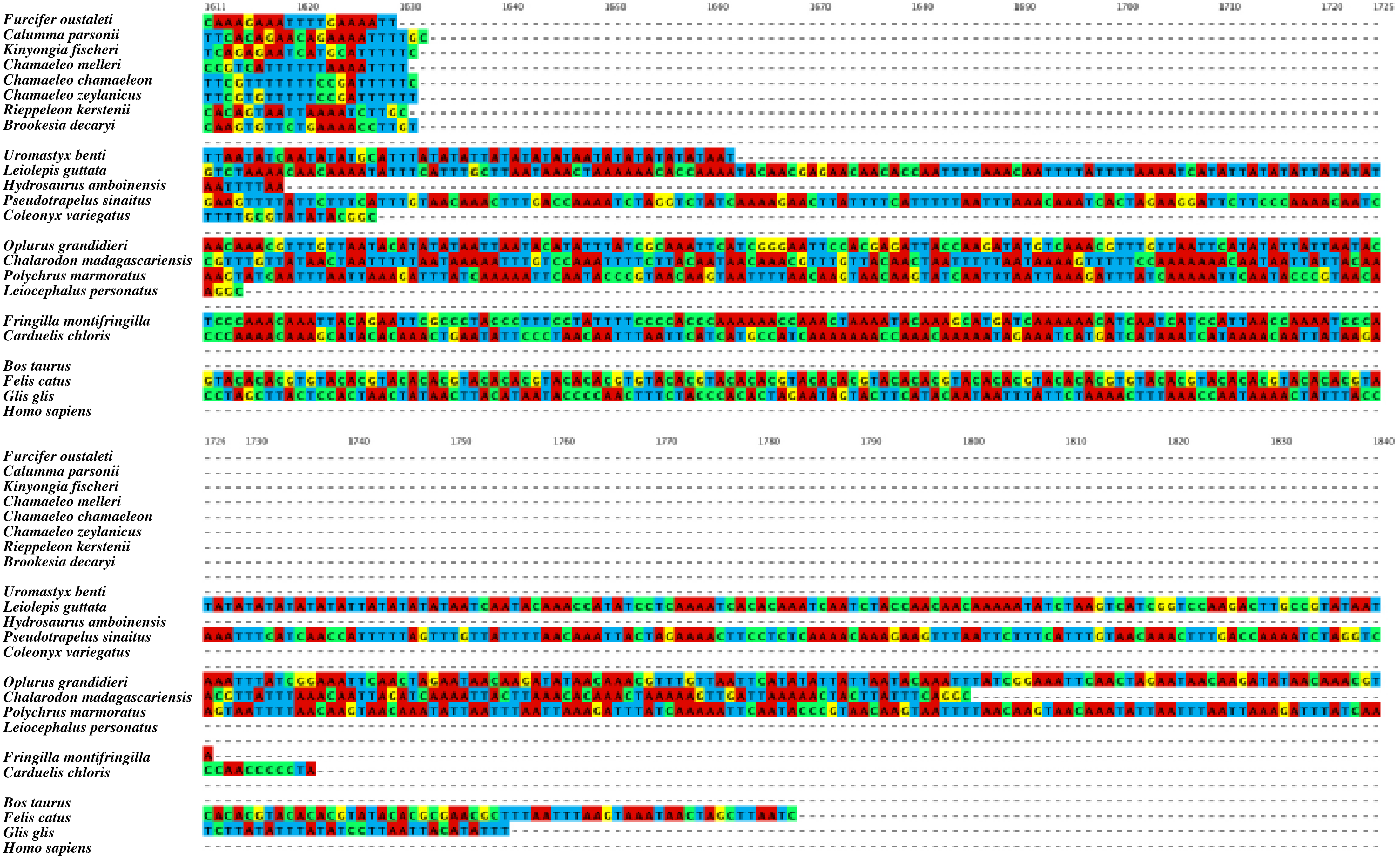
1611
1620
1630
1640
1650
1660
1670
1680
1690
1700
1710
1720
1725
Furcifer oustaleti
Calumma parsonii
Kinyongia fischeri
Chamaeleo melleri
Chamaeleo chamaeleon
Chamaeleo zeylanicus
Rieppeleon kerstenii
Brookesia decaryi
Uromastyx benti
Leiolepis guttata
Hydrosaurus amboinensis
Pseudotrapelus sinaitus
Coleonyx variegatus
Oplurus grandidieri
Chalarodon madagascariensis
Polychrus marmoratus
Leiocephalus personatus
Fringilla montifringilla
Carduelis chloris
Bos taurus
Felis catus
Glis glis
Homo sapiens
1726
1730
1740
1750
1760
1770
1780
1790
1800
1810
1820
1830
1840

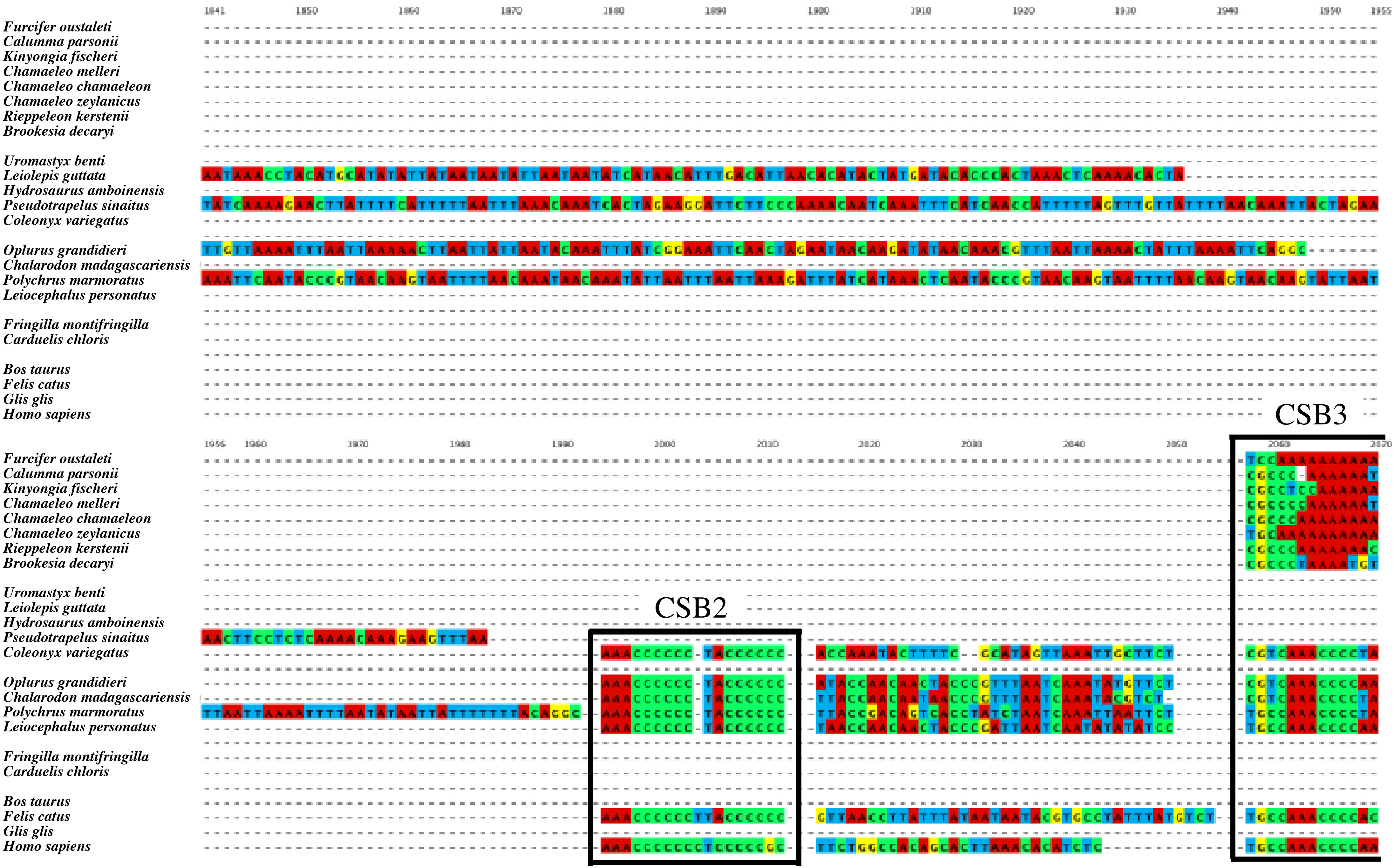
Furcifer oustaleti
Calumma parsonii
Kinyongia fischeri
Chamaeleo melleri
Chamaeleo chamaeleon
Chamaeleo zeylanicus
Rieppeleon kerstenii
Brookesia decaryi
Uromastyx benti
Leiolepis guttata
Hydrosaurus amboinensis
Pseudotrapelus sinaitus
Coleonyx variegatus
Oplurus grandidieri
Chalarodon madagascariensis
Polychrus marmoratus
Leiocephalus personatus
Fringilla montifringilla
Carduelis chloris
Bos taurus
Felis catus
Glis glis
Homo sapiens
CSB3
CSB2

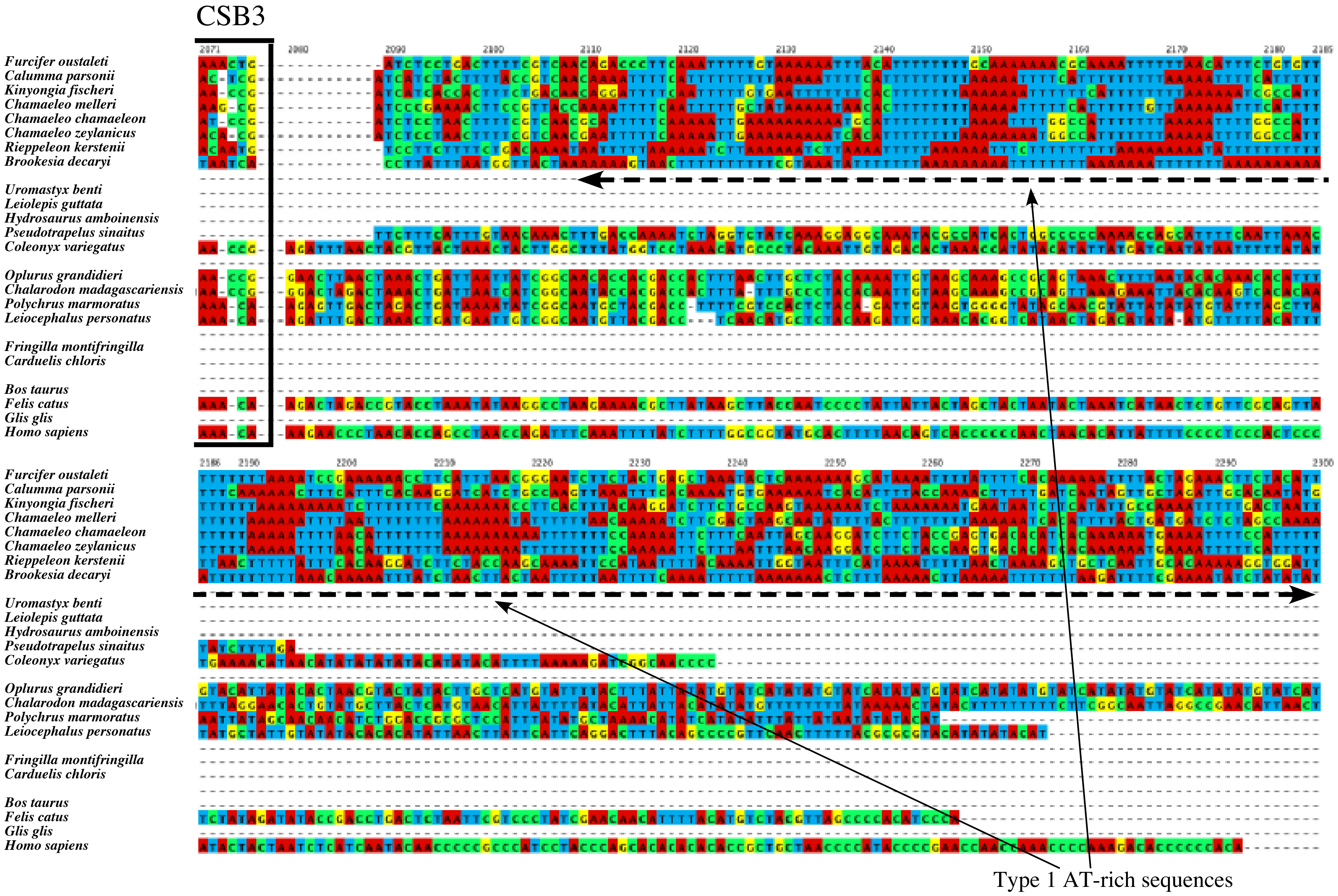
CSB3
Furcifer oustaleti
Calumma parsonii
Kinyongia fischeri
Chamaeleo melleri
Chamaeleo chamaeleon
Chamaeleo zeylanicus
Rieppeleon kerstenii
Brookesia decaryi
Uromastyx benti
Leiolepis guttata
Hydrosaurus amboinensis
Pseudotrapelus sinaitus
Coleonyx variegatus
Oplurus grandidieri
Chalarodon madagascariensis
Polychrus marmoratus
Leiocephalus personatus
Fringilla montifringilla
Carduelis chloris
Bos taurus
Felis catus
Glis glis
Homo sapiens
Type 1 AT-rich sequences

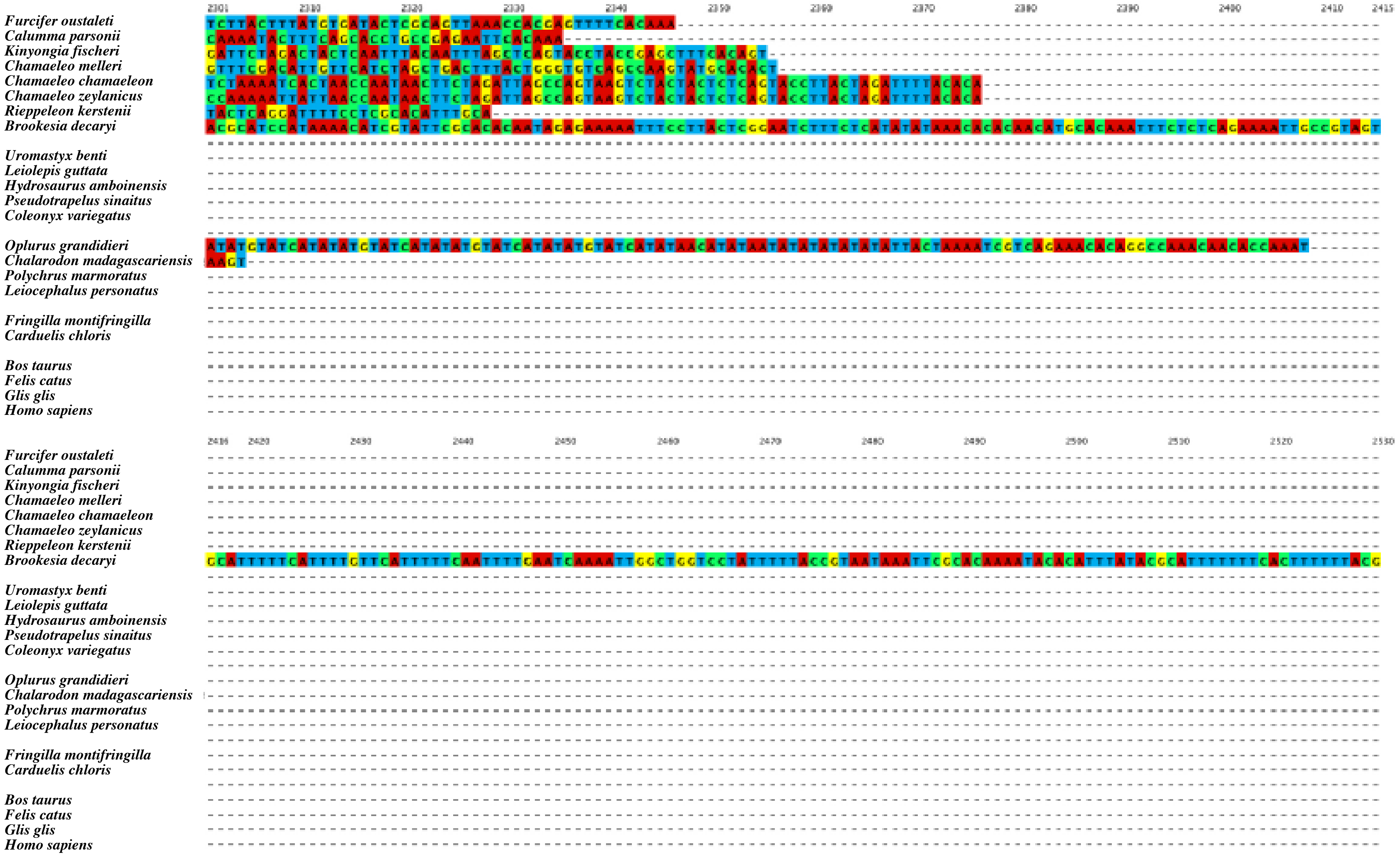
2301
2310
2320
2330
2340
2350
2360
2370
2380
2390
2400
2410
2415
Furcifer oustaleti
Calumma parsonii
Kinyongia fischeri
Chamaeleo melleri
Chamaeleo chamaeleon
Chamaeleo zeylanicus
Rieppeleon kerstenii
Brookesia decaryi
Uromastyx benti
Leiolepis guttata
Hydrosaurus amboinensis
Pseudotrapelus sinaitus
Coleonyx variegatus
Oplurus grandidieri
Chalarodon madagascariensis
Polychrus marmoratus
Leiocephalus personatus
Fringilla montifringilla
Carduelis chloris
Bos taurus
Felis catus
Glis glis
Homo sapiens
2416
2420
2430
2440
2450
2460
2470
2480
2490
2500
2510
2520
2530
Furcifer oustaleti
Calumma parsonii
Kinyongia fischeri
Chamaeleo melleri
Chamaeleo chamaeleon
Chamaeleo zeylanicus
Rieppeleon kerstenii
Brookesia decaryi
Uromastyx benti
Leiolepis guttata
Hydrosaurus amboinensis
Pseudotrapelus sinaitus
Coleonyx variegatus
Oplurus grandidieri
Chalarodon madagascariensis
Polychrus marmoratus
Leiocephalus personatus
Fringilla montifringilla
Carduelis chloris
Bos taurus
Felis catus
Glis glis
Homo sapiens

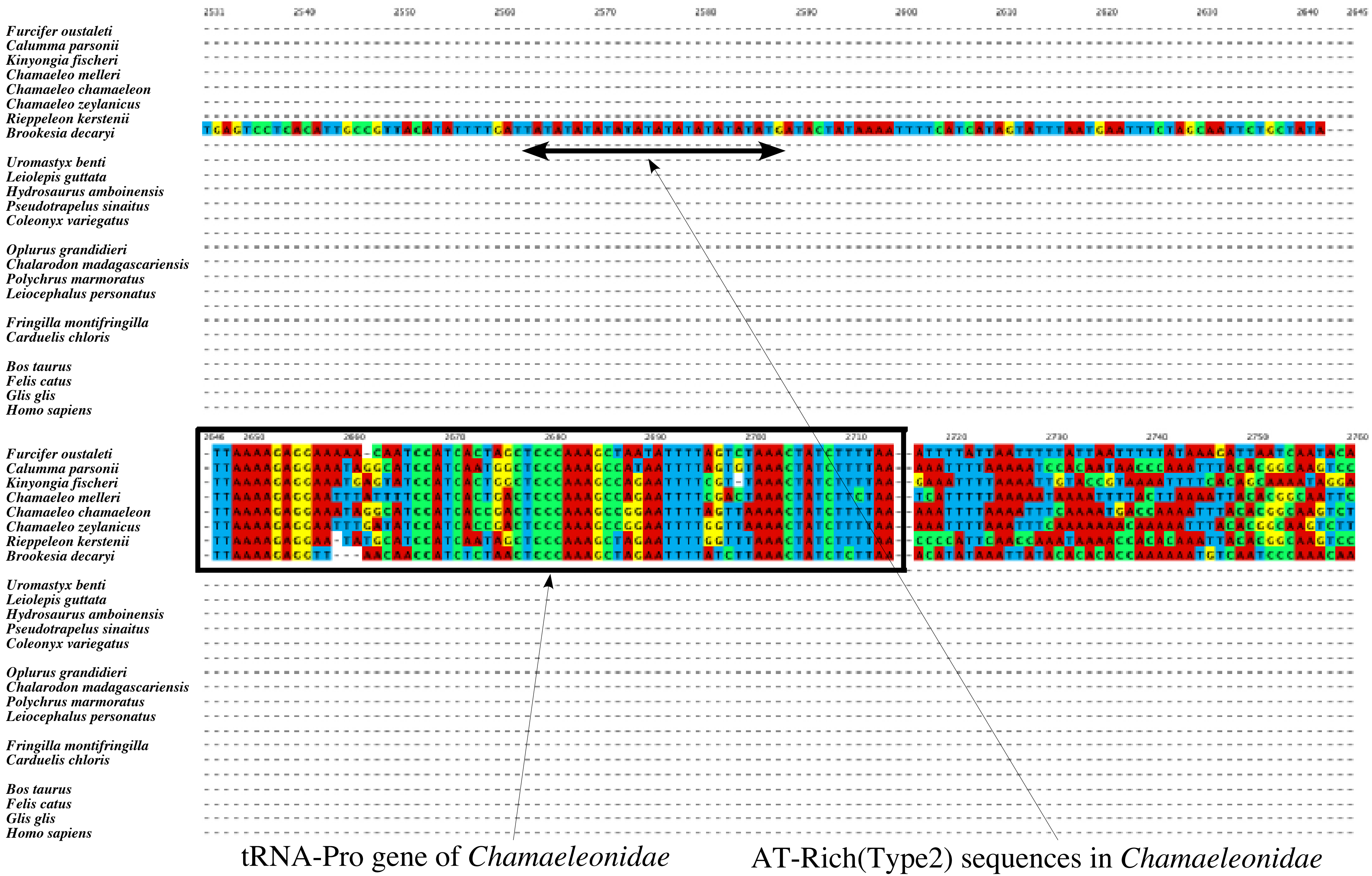
Furcifer oustaleti
Calumma parsonii
Kinyongia fischeri
Chamaeleo melleri
Chamaeleo chamaeleon
Chamaeleo zeylanicus
Rieppeleon kerstenii
Brookesia decaryi
Uromastyx benti
Leiolepis guttata
Hydrosaurus amboinensis
Pseudotrapelus sinaitus
Coleonyx variegatus
Oplurus grandidieri
Chalarodon madagascariensis
Polychrus marmoratus
Leiocephalus personatus
Fringilla montifringilla
Carduelis chloris
Bos taurus
Felis catus
Glis glis
Homo sapiens
tRNA-Pro gene of Chamaeleonidae
AT-Rich(Type2) sequences in Chamaeleonidae

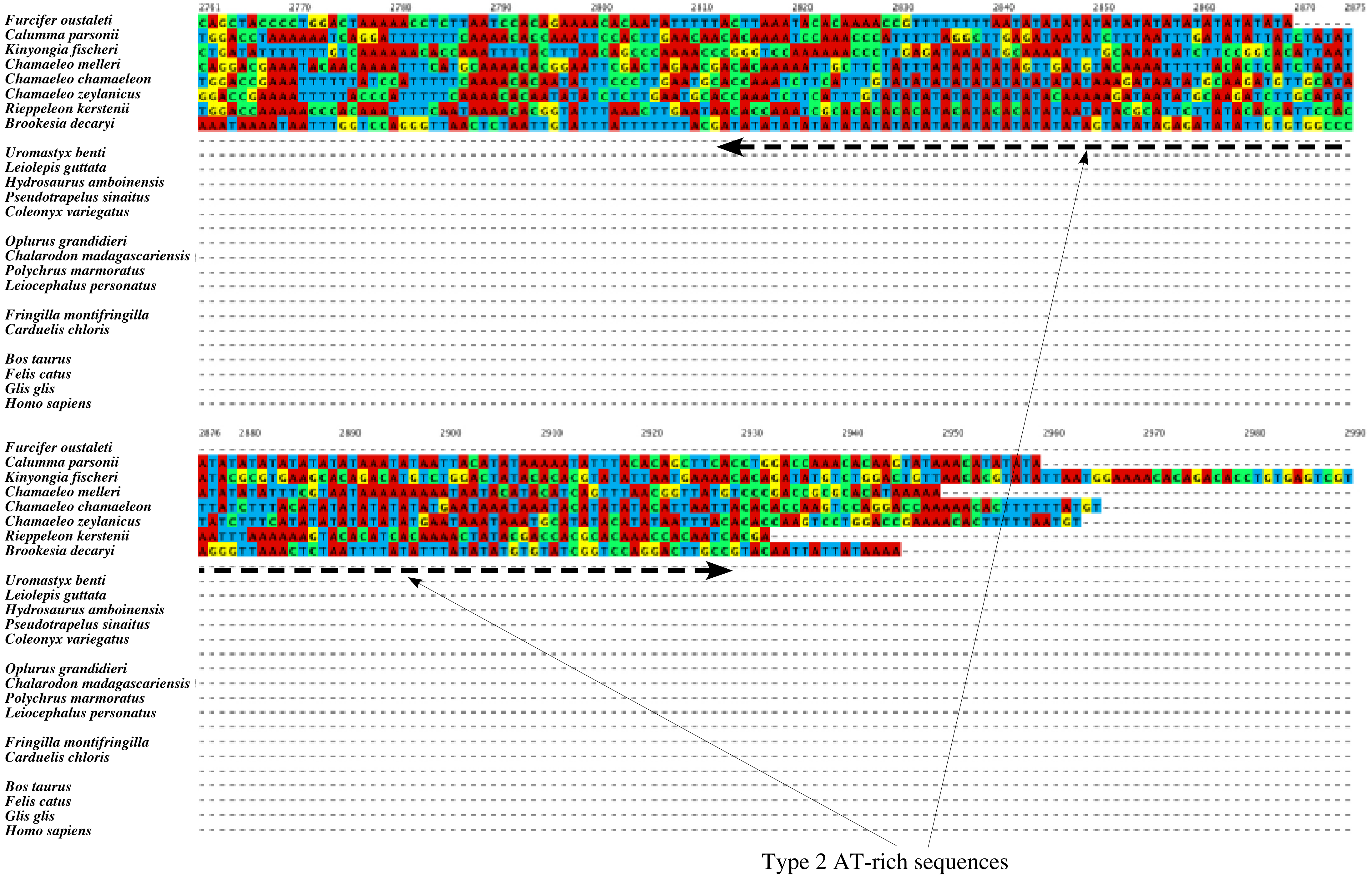
Furcifer oustaleti
Calumma parsonii
Kinyongia fischeri
Chamaeleo melleri
Chamaeleo chamaeleon
Chamaeleo zeylanicus
Rieppeleon kerstenii
Brookesia decaryi
Uromastyx benti
Leiolepis guttata
Hydrosaurus amboinensis
Pseudotrapelus sinaitus
Coleonyx variegatus
Oplurus grandidieri
Chalarodon madagascariensis
Polychrus marmoratus
Leiocephalus personatus
Fringilla montifringilla
Carduelis chloris
Bos taurus
Felis catus
Glis glis
Homo sapiens
Furcifer oustaleti
Calumma parsonii
Kinyongia fischeri
Chamaeleo melleri
Chamaeleo chamaeleon
Chamaeleo zeylanicus
Rieppeleon kerstenii
Brookesia decaryi
Uromastyx benti
Leiolepis guttata
Hydrosaurus amboinensis
Pseudotrapelus sinaitus
Coleonyx variegatus
Oplurus grandidieri
Chalarodon madagascariensis
Polychrus marmoratus
Leiocephalus personatus
Fringilla montifringilla
Carduelis chloris
Bos taurus
Felis catus
Glis glis
Homo sapiens
Type 2 AT-rich sequences

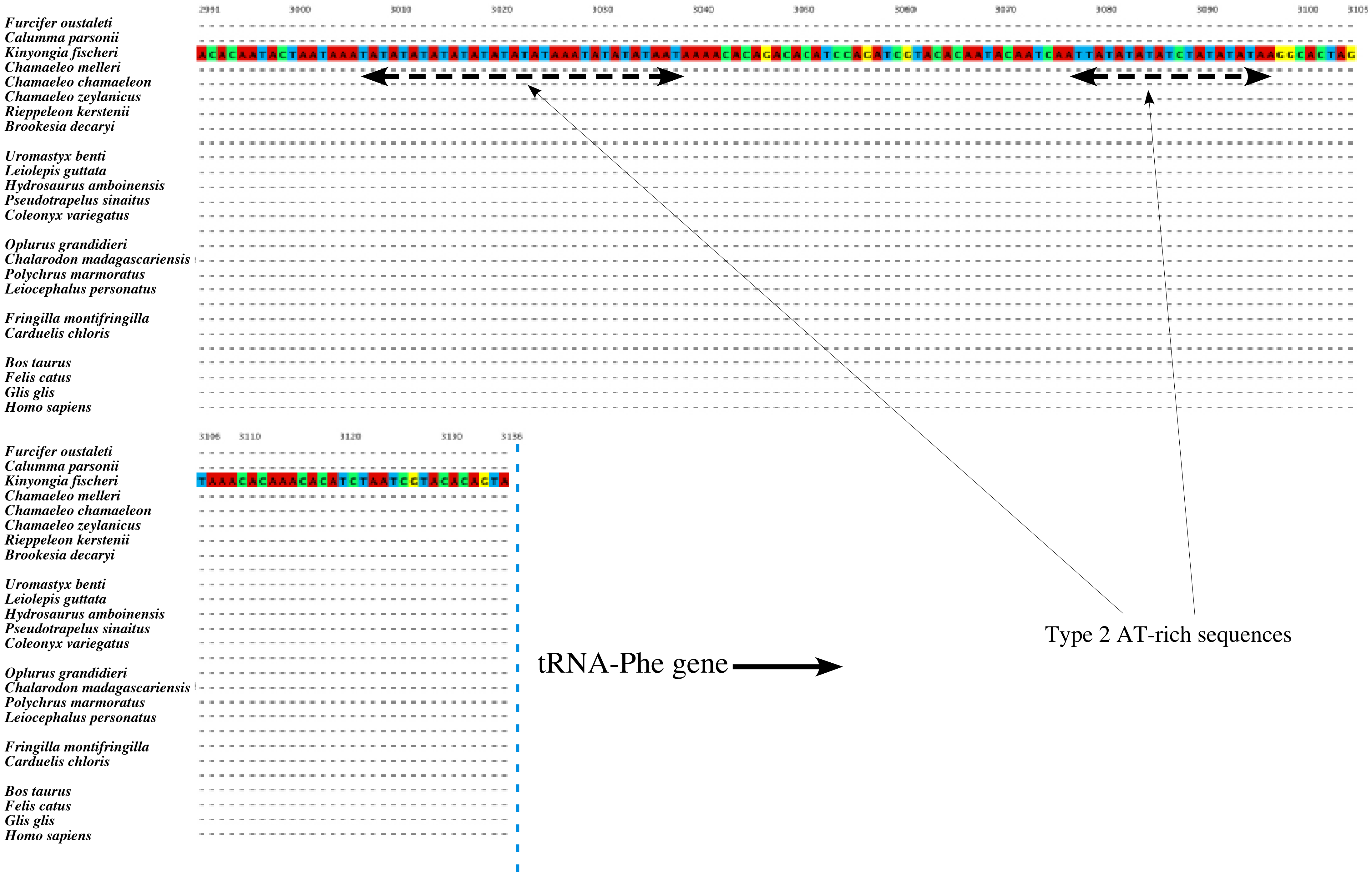
Furcifer oustaleti
Calumma parsonii
Kinyongia fischeri
Chamaeleo melleri
Chamaeleo chamaeleon
Chamaeleo zeylanicus
Rieppeleon kerstenii
Brookesia decaryi
Uromastyx benti
Leiolepis guttata
Hydrosaurus amboinensis
Pseudotrapelus sinaitus
Coleonyx variegatus
Oplurus grandidieri
Chalarodon madagascariensis
Polychrus marmoratus
Leiocephalus personatus
Fringilla montifringilla
Carduelis chloris
Bos taurus
Felis catus
Glis glis
Homo sapiens
Type 2 AT-rich sequences
tRNA-Phe gene
