# Supplementary material for: Mitochondrial genomes of acrodont lizards: timing of gene rearrangements and phylogenetic and biogeographic implications
Source: BMC Evol Biol. 2010 May 13;10:141. doi: 10.1186/1471-2148-10-141 (PMC2889956; doi:10.1186/1471-2148-10-141)
Supplement: Additional file 2 — Topological relationships assumed for the Kishino-Hasegawa and Shimodaira-Hasegawa tests in table2. [file 1471-2148-10-141-S2.PDF]

Tree1
((((((Calumma parsonii, Furcifer oustaleti), Trioceros melleri), Kinyongia fischeri), (((Chamaeleo calcaricarens, Chamaeleo chamaeleon), (Chamaeleo calyptratus, Chamaeleo zeylanicus)), Chamaeleo monachus), Chamaeleo dilepis)), Rieppeleon kerstenii), Brookesia decaryi));

Tree2
(((Calumma parsonii, Furcifer oustaleti), ((((((Chamaeleo calcaricarens, Chamaeleo chamaeleon), (Chamaeleo calyptratus, Chamaeleo zeylanicus)), Chamaeleo monachus), Chamaeleo dilepis), Trioceros melleri), Kinyongia fischeri)), (Rieppeleon kerstenii, Brookesia decaryi)));

Tree3
(((((((((Chamaeleo calcaricarens, Chamaeleo chamaeleon), (Chamaeleo calyptratus, Chamaeleo zeylanicus)),Chamaeleo monachus), Chamaeleo dilepis), Trioceros melleri), Furcifer oustaleti), Calumma parsonii), Kinyongia fischeri), Rieppeleon kerstenii), Brookesia decaryi));

Tree4
(((((((((Chamaeleo calcaricarens, Chamaeleo chamaeleon), (Chamaeleo calyptratus, Chamaeleo zeylanicus)), Chamaeleo monachus), Chamaeleo dilepis), Calumma parsonii), Kinyongia fischeri), Trioceros melleri), Furcifer oustaleti), Brookesia decaryi), Rieppeleon kerstenii));

Tree5
(((((((((Chamaeleo calcaricarens, Chamaeleo chamaeleon), (Chamaeleo calyptratus, Chamaeleo zeylanicus)), Chamaeleo monachus), Chamaeleo dilepis), Trioceros melleri), Calumma parsonii), Furcifer oustaleti), Rieppeleon kerstenii), Kinyongia fischeri), Brookesia decaryi));

Tree6
(((((Calumma parsonii, Furcifer oustaleti), Kinyongia fischeri), (((((Chamaeleo calcaricarens, Chamaeleo chamaeleon), (Chamaeleo calyptratus, Chamaeleo zeylanicus)), Chamaeleo monachus), Chamaeleo dilepis), Trioceros melleri)), Rieppeleon kerstenii), Brookesia decaryi));

Topological relationships assumed for the Kishino-Hasegawa and Shimodaira-Hasegawa tests in table 2.
