# Supplementary material for: Mitochondrial genomes of acrodont lizards: timing of gene rearrangements and phylogenetic and biogeographic implications
Source: BMC Evol Biol. 2010 May 13;10:141. doi: 10.1186/1471-2148-10-141 (PMC2889956; doi:10.1186/1471-2148-10-141)
Supplement: Additional file 3 — Non-iguanian outgroup taxa analyzed for their complete mtDNA sequence. [file 1471-2148-10-141-S3.PDF]

Non-iguanian outgroup taxa analyzed for their complete mtDNA sequence

Scientific name	Accession No.	Reference
Anguimorpha		
Shinisaurus crocodilurus	AB080274	Kumazawa 2004[1]
Abronia graminea	AB080273	Kumazawa 2004[1]
Scincomorpha		
Takydromus tachydromoides	AB080237	Kumazawa 2007[2]
Lepidophyma flavimaculatum	AB162908	Kumazawa 2007[2]
Cordylus warreni	AB079613	Kumazawa 2004[1]
Gekkota		
Gekko vittatus	AB178897	Kumazawa 2007[2]
Coleonyx variegates	AB114446	Kumazawa 2007[2]
Amphisbaenia		
Geocalamus acutus	AB162909	Kumazawa 2007[2]
Sphenodontida		
Sphenodon punctatus	AF534390	Rest et al. 2003[3]
Aves		
Gallus gallus	X52392	Desjardins and Morais 1990[4]
Crocodylia		
Alligator mississippiensis	Y13113	Janke and Arnason 1997[5]
Testudines		
Chelonia mydas	AB012104	Kumazawa and Nishida 1999[6]
Mammalia		
Bos taurus	J01394	Anderson et al. 1982[7]
Ornithorhynchus anatinus	X83427	Janke et al. 1996[8]
Amphibia		
Xenopus laevis	M10217	Roe et al. 1985[9]
Typhlonectes natans	AF154051	Zardoya and Meyer 2000[10]
Osteichthyes		
Protopterus dolloi	L42813	Zardoya and Meyer 1996[11]
Latimeria chalumnae	U82228	Zardoya and Meyer 1997[12]

[1]DNA Res. 11:137-144

[2]Gene 388:19-26

[3]Mol. Phylogenet. Evol. 29:289-297

[4]J. Mol. Biol. 212:599-634

[5]Mol. Biol. Evol. 14:1266-1272

[6]Mol. Biol. Evol. 16:784-792

[7]J. Mol. Biol. 156:683-717

[8]Proc. Natl. Acad. Sci. USA 94:1276-1281

[9]J. Biol. Chem. 260:9759-9774

[10]Genetics 155:765-775

[11]Genetics 142:1249-1263

[12]Genetics 146:995-1010
