# Supplementary material for: Mitochondrial genomes of acrodont lizards: timing of gene rearrangements and phylogenetic and biogeographic implications
Source: BMC Evol Biol. 2010 May 13;10:141. doi: 10.1186/1471-2148-10-141 (PMC2889956; doi:10.1186/1471-2148-10-141)
Supplement: Additional file 4 — A Bayesian tree reconstructed using mitogenomic nucleotide sequences by including the tuatara and retaining gap-containing sites (49 taxa, 9689 sites). [file 1471-2148-10-141-S4.PDF]

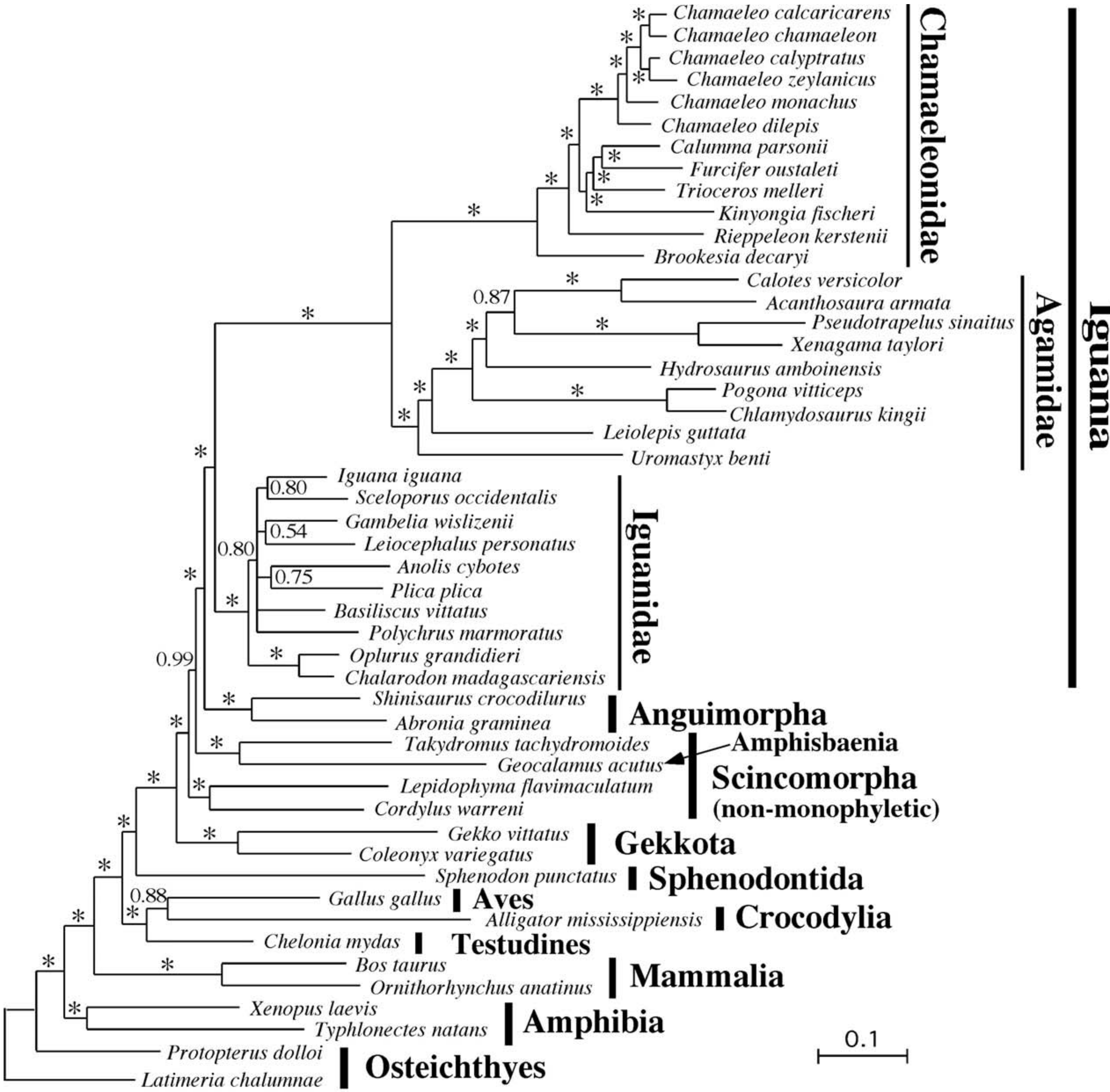

A Bayesian tree reconstructed using mitogenomic nucleotide sequences by including the tuatara and gap-containing sites (49 taxa, 9689 sites). Values shown are the Bayesian posterior probabilities and an asterisk stands for 1.00. See table 1 and Additional File 3 for accession numbers of mitogenomic data for individual taxa.
